# A *Caenorhabditis elegans* protein with a PRDM9-like SET domain localizes to chromatin-associated foci and promotes spermatocyte gene expression, sperm production and fertility

**DOI:** 10.1371/journal.pgen.1007295

**Published:** 2018-04-27

**Authors:** Christoph G. Engert, Rita Droste, Alexander van Oudenaarden, H. Robert Horvitz

**Affiliations:** 1 Dept. Biology, MIT, Cambridge, MA, United States of America; 2 Howard Hughes Medical Institute, MIT, Cambridge, MA, United States of America; 3 Computational & Systems Biology Graduate Program, MIT, Cambridge, MA, United States of America; 4 Dept. Physics, MIT, Cambridge, MA, United States of America; University of Cambridge, UNITED KINGDOM

## Abstract

To better understand the tissue-specific regulation of chromatin state in cell-fate determination and animal development, we defined the tissue-specific expression of all 36 *C*. *elegans* presumptive lysine methyltransferase (KMT) genes using single-molecule fluorescence *in situ* hybridization (smFISH). Most KMTs were expressed in only one or two tissues. The germline was the tissue with the broadest KMT expression. We found that the germline-expressed *C*. *elegans* protein SET-17, which has a SET domain similar to that of the PRDM9 and PRDM7 SET-domain proteins, promotes fertility by regulating gene expression in primary spermatocytes. SET-17 drives the transcription of spermatocyte-specific genes from four genomic clusters to promote spermatid development. SET-17 is concentrated in stable chromatin-associated nuclear foci at actively transcribed *msp* (major sperm protein) gene clusters, which we term *msp* locus bodies. Our results reveal the function of a PRDM9/7-family SET-domain protein in spermatocyte transcription. We propose that the spatial intranuclear organization of chromatin factors might be a conserved mechanism in tissue-specific control of transcription.

## Author summary

During animal development, the generation of diverse cell types, such as nerve cells or muscle cells, requires differential gene expression. Lysine methyltransferases (KMTs) help determine which regions of the genome and hence which genes are expressed in each cell type. To enable the study of KMT function in development, Engert et al. defined the spatial expression patterns of all 36 presumptive KMT genes in the nematode *Caenorhabditis elegans*. While one-third of KMT genes were expressed broadly, the expression of most KMT genes was restricted to only certain cell types. The KMT gene *set-17* was expressed in sperm-producing cells, where it proved to be necessary for normal fertility. The SET domain of the SET-17 protein is similar to the SET domain of the mammalian KMT PRMD9, which functions in fertility in mammals; SET-17 lacks the other domains of PRDM9. The authors showed that *set-17* promotes fertility in sperm cell precursors by enabling the expression of clusters of genes that are required for the generation of sperm. Unexpectedly, the SET-17 protein localized to distinct spatial regions in the nuclei of sperm cell precursors. The authors propose that the spatial organization of KMTs and other factors that control gene expression is a broadly used mechanism in cell-type-specific gene regulation.

## Introduction

Chromatin state can be regulated at the tissue-specific and cell-type-specific levels. Both active transcription and gene silencing require chromatin regulatory mechanisms to render DNA accessible or inaccessible, respectively. The post-translational modification of specific lysine residues in the tails of histone proteins by methylation or acetylation is an important mechanism of chromatin regulation [1,2]. Histone lysine methylation can determine cell-type-specific gene expression states [3], and broad histone methylation domains at promoters are necessary for cell-fate maintenance; misregulation of such domains can drive oncogenic states [4,5]. Lysine methylation of histone tails is predominantly catalyzed by the SET domains of lysine methyltransferases (KMTs), and the SET domain can be used to identify putative lysine methyltransferases across eukaryotic genomes [6–9]. Fifty presumptive KMTs are encoded by the human genome, most of which remain uncharacterized biochemically or functionally.

While extensive analysis of histone methylation profiles in specific cell-types has revealed tissue-specific chromatin regulation, it has been difficult to determine the tissue-specificity of KMT function. In mammals, PRDM-type KMTs have emerged as tissue-specific chromatin regulators. The founding member, PRDM1, was identified as a master transcription factor in hematopoietic differentiation [10]. PRDM9 functions in the determination of meiotic recombination sites in the mammalian germline [11]. PRDM3 and PRDM16 are required for heterochromatin formation in adipose tissues [12]. The SET-domain families of PRDM-type KMTs are named according to their mammalian representatives and are broadly conserved across metazoans. However, domains outside the SET-domain can vary by organism. For example, whereas *C*. *elegans* BLMP-1 and its mammalian ortholog PRDM1 share both SET and Zn-finger domains, SET-17 (which is the only other PRDM-type KMT in *C*. *elegans*) does not share the KRAB or Zn-finger domains of its mammalian SET-domain family counterparts PRDM9 and PRDM7 (S1A and S2B Figs).

The organization of the genome into spatial domains is emerging as an important factor in the specification of cell-fate [13]. These domains can facilitate the physical interaction of enhancers with target promoters [14] and can define heterochromatin domain boundaries [15]. Domain organization of the genome is important in development and disease [16]. The relationship between tissue-specific chromatin signatures, spatial domains and transcriptional regulation remains poorly understood [17,18].

The *C*. *elegans* genome encodes 36 presumptive KMTs, most of which are conserved in mammals based on their SET domain sequences (S1A Fig). Many of these KMTs remain uncharacterized. Previous analysis of *C*. *elegans* KMT mutants showed that loss of most individual KMTs does not affect embryonic development or post-embryonic vulval development [9]. Our goal was to define the tissue-specific expression patterns of all *C*. *elegans* KMTs and focus functional analyses on a specific tissue.

## Results

### Map of endogenous KMT mRNA expression reveals both tissue-specificity and abundant KMT expression in the germline

To analyze the tissue-specificity of KMTs in *C*. *elegans*, we determined the endogenous KMT mRNA expression patterns for all 36 presumptive KMT genes using single-molecule fluorescence *in situ* hybridization (smFISH) [19]. smFISH allows the detection of endogenous mRNA in all *C*. *elegans* tissues, including the germline, in which multi-copy transgenes are silenced [20].

**Fig 1:**
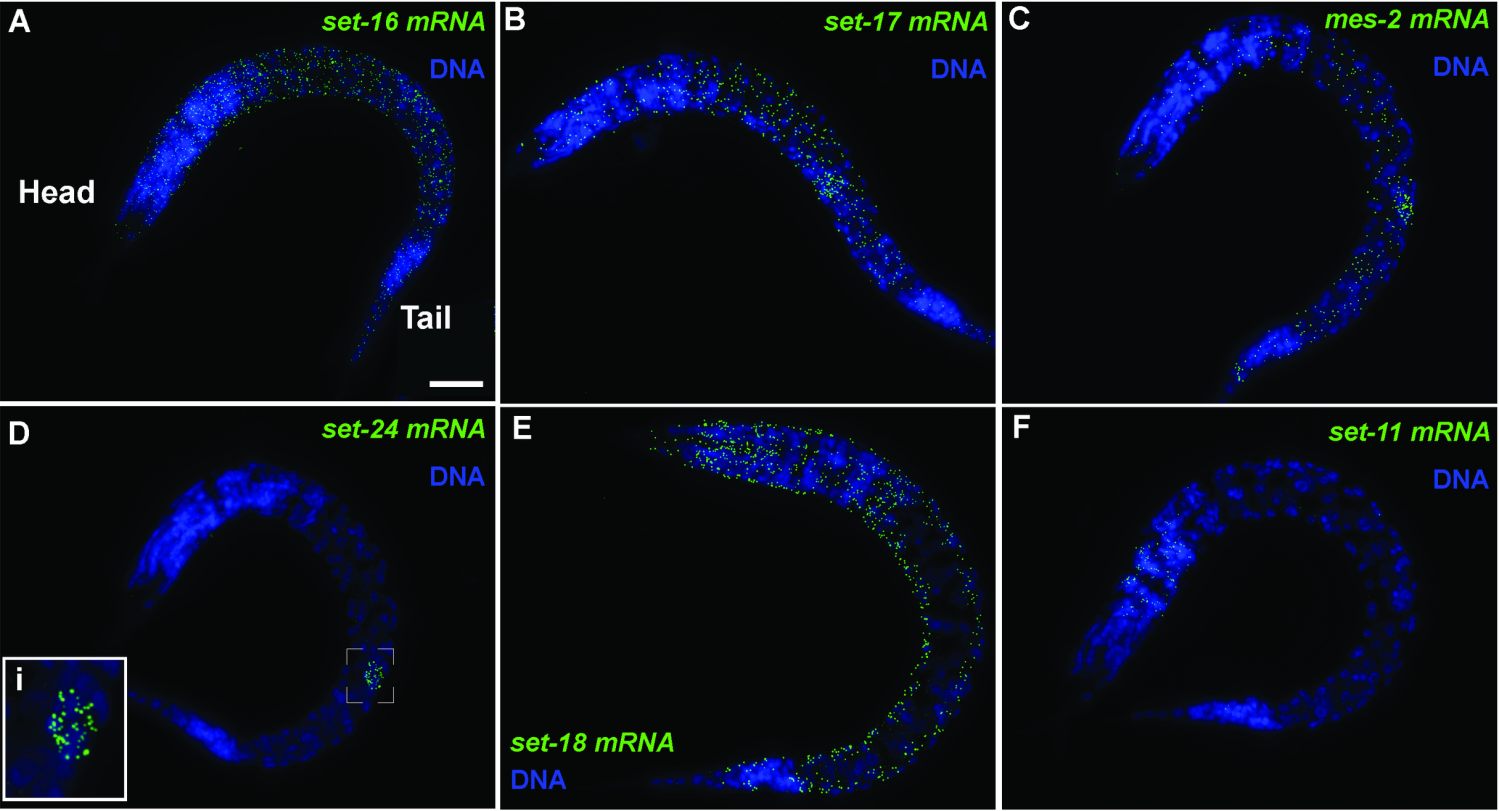
Endogenous expression of lysine methyltransferase mRNAs by smFISH in whole *C*. *elegans*. Endogenous mRNA expression of transcripts in whole L1 larval stage *C*. *elegans* hermaphrodites of (A) ubiquitous *set-16*, (B) broadly-expressed *set-17*, (C) hypodermal and germline *mes-2*, (D) germline-specific *set-24*, (E) muscle-specific *set-18* and (F) neuron-specific *set-11*. Scale bar, 10 μm. (D) (i) Inset of the primordial germ cells expressing *set-24* mRNA.

**Fig 2:**
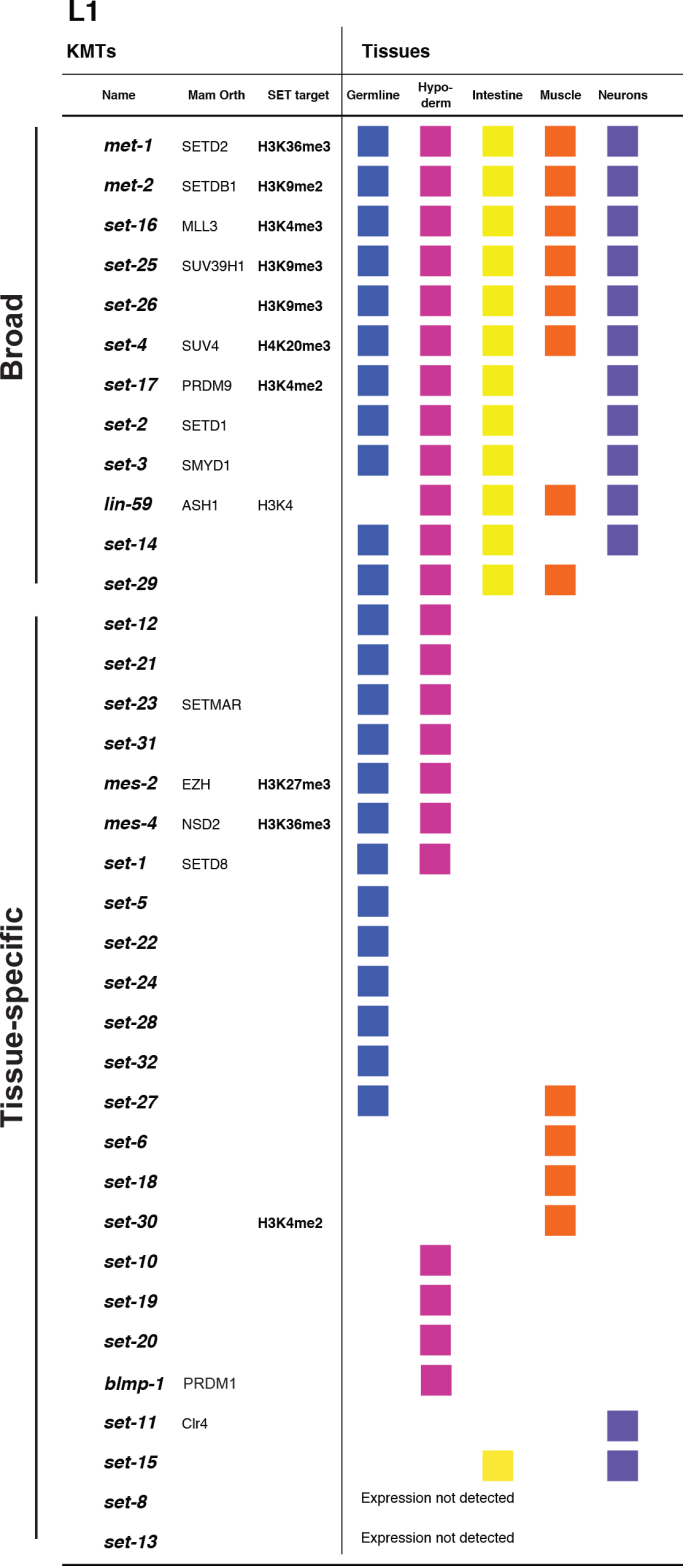
L1 tissue-specific expression patterns of all *C*. *elegans* KMT genes. Most KMT gene were expressed with tissue-specificity. Expression in a tissue was defined as more than one transcript detected in multiple cells in all animals. Mam Orth, mammalian ortholog of the *C*. *elegans* protein; SET target, experimentally shown to catalyze histone lysine methylation at these targets.

The KMT gene expression patterns in the L1 larval stage fell into two categories, broad and tissue-specific: 12 KMTs were expressed broadly, while 22 were detectable in only one or two tissues (Figs 1 and 2). No expression was detected for two KMTs. The 12 broadly expressed KMTs included the *C*. *elegans* orthologs of general chromatin modifiers *set-16* (MLL; Figs 1A and 2), *met-*1 (SETD2), *met-2* (SETDB1), *set-25* (SUV39) and *set-4* (SUV420). The SET-domain orthologs of some KMTs that function tissue-specifically in mammals were also broadly expressed, such as *set-17*, the SET domain of which is similar to the SET domains of the PRDM9/7 family (Figs 1B, S1A and S3B). *mes-2*, the *C*. *elegans* ortholog of the Polycomb KMT EZH, was expressed in only hypoderm and germline (Fig 1C). We verified the expression pattern of *mes-*2, and that of *mes-4*, with two independently designed smFISH probe-sets. Consistent with our findings, recent RNAseq studies of single postembryonic *C*. *elegans* cells also have reported somatic expression of *mes-2* and *mes-*4 [21]. Twelve KMTs were detectable in only a single tissue: five were germline-specific (e.g. Fig 1D), three muscle-specific (e.g. Fig 1E), three hypoderm-specific and *set-11* was neuron-specific (Fig 1F). Our data show that at least 11 KMTs were expressed in any given tissue, and the germline was the tissue with the broadest KMT expression. We conclude that most KMTs are expressed tissue-specifically, although expression of 12 KMTs occurs in most cells.

### SET-17 functions in the germline to promote sperm production and fertility

**Fig 3:**
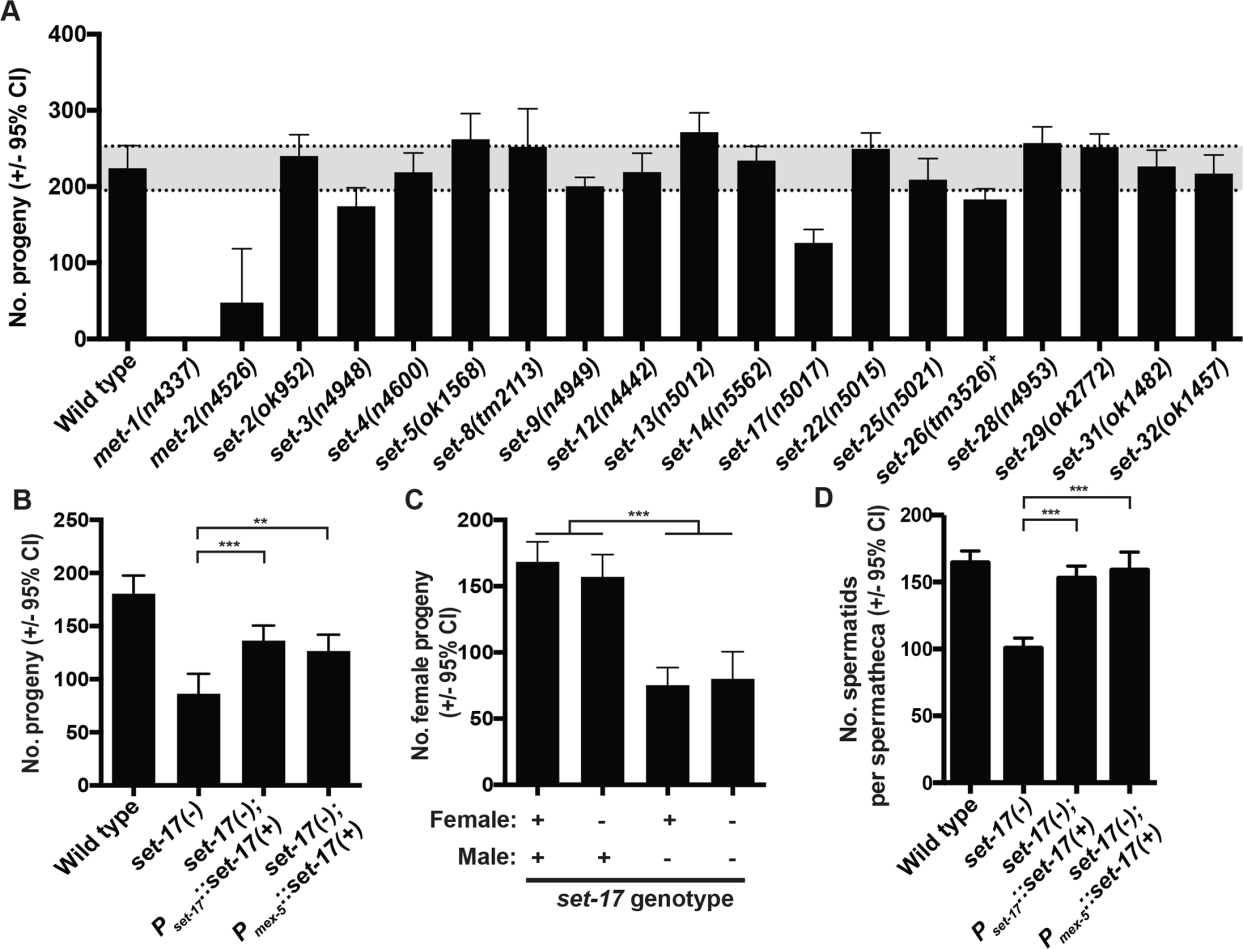
*set-17* functions in the germline to promote sperm-production and fertility. A) Broodsizes of mutants defective in KMT genes expressed in the germline. Progeny number was determined for single adult hermaphrodites over three days and progeny were scored as adults after 3–6 days. Grey shaded area indicates the range of the wild-type 95% confidence interval. n > 10 for all genotypes. *set-23*, *set-24* and *set-27* mutants were not available, and instead these genes were examined using RNAi treated wild-type animals; fertility was not affected. B) Broodsize of wild-type, *set-17*, *set-17; P*_*set-17*_::*set-17(+)* (expressing wild-type *set-17* from its endogenous promoter) and *set-17; P*_*mex-5*_::*set-17(+)* (expressing wild-type *set-17* from the *mex-5* germline-specific promoter) hermaphrodites. n > 20; *** P < 0.0001, ** P < 0.0012, t-test. C) Progeny of mating single males (wild-type or *set-17* mutant) and single females (wild-type or *set-17* mutant). Strains carried the *fog-2(q71)* mutation, which feminizes hermaphrodites by suppressing hermaphrodite but not male sperm production and ensured that all progeny were cross progeny. After 24 hr the male was removed, and the female allowed to lay progeny until completion. Females were placed on fresh plates every 24 hr over 4 days. Cross progeny were scored as number of adult female progeny 3–7 days after mating; only females were scored because adult males burrow, crawl off plates and clump together, making their quantification unreliable. n > 15. *** P < 0.0001, t-test. D) Spermatid counts in individual spermathecas of the indicated genotypes. Spermatid counts were determined by imaging DAPI-stained spermatid nuclei in hermaphrodites fixed in 4% formaldehyde 12 hr post-L4 around the time of first fertilization. n: wild-type = 26, *set-17(-)* = 32, *set-17(-);* P_*set-17*_::*set-17(+)* = 33, *set-17(-);* P_*mex-5*_::*set-17(+)* = 15. *** P < 0.0001, t-test.

To assess the functions of individual KMTs, we focused on germline-expressed KMTs. The mRNAs of all KMTs that were detectable in primordial germ cells in the L1 were also detectable in the adult germline (S1B Fig, S2 Fig). The primary measure of germline function is fertility. We therefore determined the number of progeny of all available and viable mutants of germline-expressed KMTs (Fig 2, Fig 3A). All of the mutations we tested were out-of-frame deletions and therefore likely to cause complete loss of function. It had been previously reported that the fertility of *met-1* and *met-2* mutants declines over several generations at elevated temperatures [9]. To avoid such effects, we maintained all strains at lower temperatures and raised the temperature only for fertility measurements. Our assay confirmed the previously reported fertility defects of *met-1* and *met-2* mutants [9], while animals with individual mutations of most germline-expressed KMTs exhibited normal fertility.

*set-17(n5017)* mutants showed a 50% reduction in fertility (Fig 3A and 3B). *set-17(n5017)* deletes the first 135 amino acids of *set-17*, including part of the SET-domain (S3A Fig). A second allele, *set-17(gk417488)*, an ochre termination codon at amino acid 84, caused a fertility defect similar to that of *set-17(n5017)* (hereafter *set-17*, S3A Fig). Expression of wild-type *set-17* partially rescued the fertility defect of *set-17* mutants when expressed as a single-copy insertion [22,23] from its endogenous promoter (P_*set-17*_) or expressed in the germline from a germline-specific promoter (P_*mex-5*_, Fig 3B). We found that the expression of *set-17* was restored only partially by either the *set-17* endogenous promoter (58% of the wild-type level) or the germline-specific *mex-5* promoter (47% of the wild-type level) (S6B and S6F Fig). This partial restoration of *set-17* expression could account for the partial rescue of fertility. Mutations in the other germline-expressed KMTs we tested did not affect the fertility defect of *set-17* mutants (S3C Fig). We conclude that loss of *set-17* function reduces fertility and that *set-17* function in the germline is sufficient to promote fertility.

*set-17* could function in either the sperm- or the oocyte-producing germline, or both, to promote fertility. To distinguish among these possibilities, we conducted mating experiments with *set-17* males and females. We feminized *set-17* hermaphrodites using the *fog-2(q71)* mutation, which suppresses sperm production in hermaphrodites [24]. If and only if males were mutant for *set-17* was there a reduction in the number of cross-progeny (Fig 3C): when mated to wild-type males, *set-17* females produced the same number of progeny as wild-type females; by contrast, both wild-type and *set-17* females displayed a 50% reduction in the number of progeny when mated to *set-17* males. We conclude that SET-17 functions in only the male germline to promote fertility.

In *C*. *elegans* spermatogenesis, germ cells differentiate into primary spermatocytes while they are undergoing meiosis I. One primary spermatocyte then differentiates into a secondary spermatocyte, which generates four haploid spermatids with the completion of meiosis II. Spermatids are then stored in the spermatheca of the hermaphrodite or the male. Before fertilization spermatids are activated by external cues from oocytes to become actively crawling spermatozoa. To determine if *set-17* functions in spermatid production, we counted the number of spermatids in the spermatheca of young adult hermaphrodites after spermatid production had concluded. *set-17* mutants exhibited a 50% reduction in the number of spermatids per spermatheca (Fig 3D). This defect was rescued by expressing wild-type *set-17* either from its endogenous promoter or germline-specifically. We did not detect functional abnormalities in *set-17* spermatozoa in an *in vitro* activation assay or when monitoring *set-17* spermatozoa crawling from the uterus to the spermatheca after mating, suggesting that *set-17* spermatids function normally once produced. We conclude that *set-17* functions in spermatid production to promote fertility.

### SET-17 promotes the production of fibrous-body membranous organelles in primary spermatocytes

**Fig 4:**
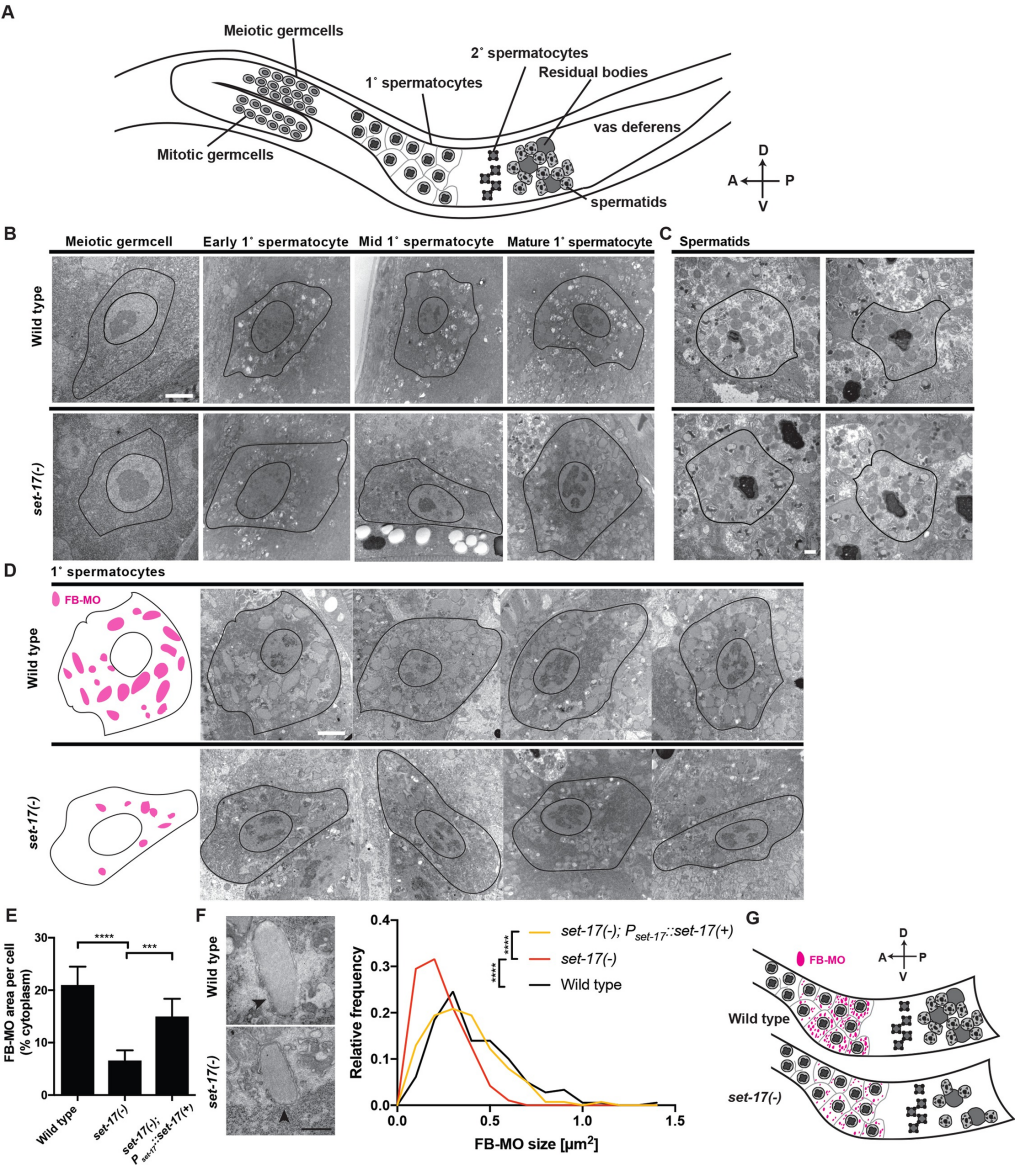
*set-17* promotes the number and size of major sperm protein vesicles in primary spermatocytes. A) Schematic of the male germline. Meiotic germ cells differentiate into primary spermatocytes, which mature as they progress along the gonad into secondary spermatocytes and then spermatids. A, anterior; P, posterior; D, dorsal; V, ventral. B) Electron micrographs of spermatocyte differentiation in wild-type and *set-17* males. All spermatocyte differentiation stages are present in *set-17* mutants. Images were staged based on their relative positions along the germline. Scale bar, 2 μm. C) Electron micrographs of spermatids in wild-type and *set-17* males. Spermatids appear normal in *set-17* mutants. Scale bar, 500 nm. D) Electron micrographs of mature primary spermatocytes from wild-type and *set-17* males. Left, schematic traces of the outlines of the left-most panels of wild-type and *set-17* primary spermatocytes (black), respectively, indicating the areas and positions of FB-MOs (magenta). Fibrous-body membranous organelles (FB-MOs) are smaller in *set-17* mutants. Scale bar, 2 μm. E) Percent cytoplasmic cross-sectional area taken up by FB-MOs per cell in wild-type, *set-17* and *set-17; P*_*set-17*_::*set-17(+)* male mature primary spermatocytes. n = 10; **** P < 0.0001, *** P < 0.001, t-test. F) Frequency distributions of FB-MO cross-sectional areas (size) in wild-type, *set-17* and *set-17; P*_*set-17*_::*set-17(+)* male primary spermatocytes. Arrowheads, FB-MOs in micrographs of representative wild-type and *set-17* spermatocytes. n > 95; **** P < 0.0001, KS-test; Mean: wild-type = 0.41 μm^2^, *set-17* = 0.24 μm^2^, *set-17; P*_*set-17*_::*set-17(+)* = 0.35 μm^2^; Scale bar, 500 nm. G) Schematic of sperm production in wild-type and *set-17* males. In *set-*17 mutants, defective FB-MO production in primary spermatocytes leads to smaller FB-MOs and a reduction in the production of otherwise normal spermatids. A, anterior; P, posterior; D, dorsal; V, ventral.

To identify morphological abnormalities that might be related to the decreased spermatid production and fertility of *set-17* mutants, we used electron microscopy to examine the stages of spermatocyte development and spermatid formation (Fig 4A) in *set-17* males. Although the gross morphologies of both spermatocytes and spermatids appeared normal (Fig 4B and 4C), the cytoplasm of mature *set-17* primary spermatocytes displayed a striking ultrastructural abnormality. Fibrous-body membranous organelles (FB-MOs, Fig 4D–4F), which store paracrystals of the major sperm proteins (MSPs)[25], defined relatively little of the cytoplasmic area of *set-17* primary spermatocytes. MSPs are produced in spermatocytes and function in spermatozoa, in which they form the pseudopod that drives motility. We found that FB-MOs covered about 20% of the cross-sectional cytoplasmic area of wild-type primary spermatocytes and only about 8% of the cytoplasmic area in *set-17* spermatocytes (Fig 4E). This abnormality was rescued in *set-17* males that expressed wild-type *set-17* as a transgene driven by the endogenous *set-17* promoter (Fig 4E). We found that the reduction in overall FB-MO cross-sectional area in *set-17* spermatocytes was caused by a reduction of individual FB-MO cross-sectional area to half of that in wild-type spermatocytes (Fig 4F). *set-17* males were normal in their number of FB-MOs (S4A and S4B Fig, Methods). The reduction of individual FB-MO area in *set-17* spermatocytes leads us to conclude that *set-17* promotes FB-MO production in primary spermatocytes. We suggest that an absence of *set-17* function causes a reduction of FB-MO production in spermatocytes. We speculate that reduced FB-MO production contributes to reduced spermatid number that in turn leads to reduced fertility (Fig 4G).

### SET-17 is expressed in primary spermatocytes and localizes to nuclear, chromatin-associated foci

**Fig 5:**
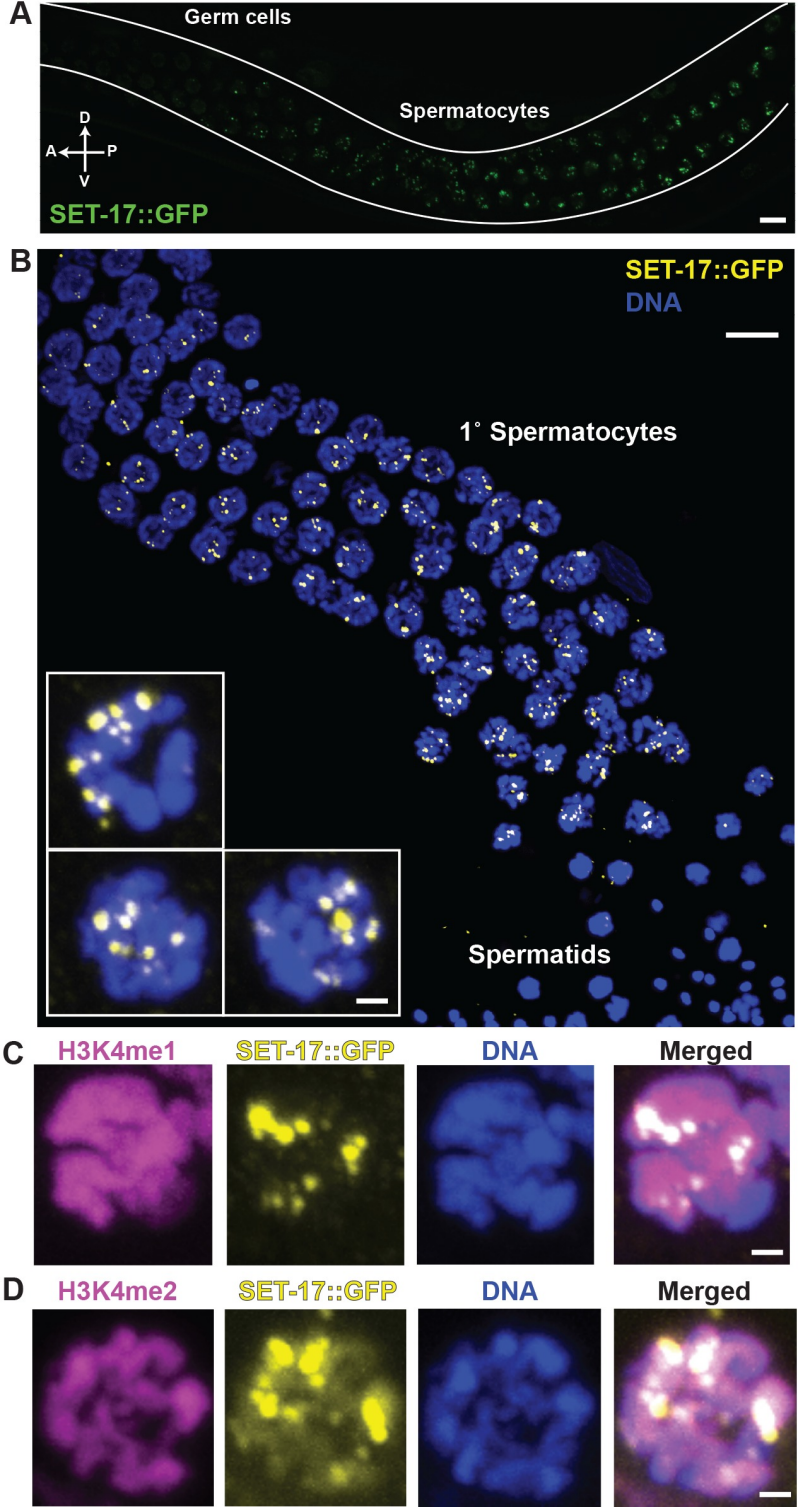
SET-17 is expressed in the nuclei of primary spermatocytes and enriched in chromatin-associated foci. A) Confocal imaging of a longitudinal section showing SET-17::GFP expression in germ cells and spermatocytes of an adult male germline. White lines delineate the gonad. Scale bar, 10 μm. A, anterior; P, posterior; D, dorsal; V, ventral. B) Representative Z-axis projection of confocal images of extruded and formaldehyde-fixed male germlines expressing SET-17::GFP and stained for GFP by immunofluorescence (yellow). DNA stained with DAPI (blue). Scale bar, 10 μm. Inserts, higher magnification views of individual representative nuclei. Scale bar, 1 μm. C) & D) Representative Z-axis projection of primary spermatocyte nuclei of males expressing SET-17::GFP. Nuclei were stained by immunofluorescence for GFP and H3K4me1 C) or H3K4me2 D), respectively. DNA, DAPI. Scale bar, 1 μm.

To determine the expression pattern and subcellular localization of SET-17 protein, we used a single-copy transgene that expresses SET-17::GFP under the *set-17* endogenous promoter and can rescue the fertility, sperm-production and FB-MO-production defects of *set-17* mutants (Figs 3C, 3E and 4E). Using confocal microscopy, we examined immobilized live male germlines (Fig 5A) and fixed male germlines that were extruded from adult males by microsurgery and labeled by immunofluorescence (Fig 5B). We found that in the male germline SET-17::GFP was expressed predominantly in primary spermatocytes (Fig 5A and 5B). SET-17::GFP was first detectable at the initiation of spermatocyte differentiation in L4 and adult males. The SET-17::GFP level increased with the stage of spermatocyte progression and peaked in late-stage primary spermatocytes (Fig 5A and 5B). Strikingly, SET-17::GFP localized to foci in the nuclei of primary spermatocytes (Fig 5B i-iii). The SET-17 foci were associated with DNA, ranged from 4–14 per primary spermatocyte and increased in number with overall SET-17::GFP expression (S5A Fig). In hermaphrodites, SET-17::GFP was similarly expressed in primary spermatocytes and localized to nuclear foci (S5B Fig). In hermaphrodites, spermatid production occurs during the L4 stage. We also detected SET-17::GFP expression during oocyte production (S5C Fig), even though SET-17 does not obviously function in oocytes for fertility (Fig 3C). In hypodermal nuclei, the distribution of SET-17::GFP was pan-nuclear (S5D Fig), suggesting that SET-17 localization to foci is related to the function of SET-17 in spermatocytes. The SET-17::GFP expression pattern is consistent with our conclusion that SET-17 functions in primary spermatocytes and suggests that SET-17 functions in nuclei in chromatin-associated foci.

To investigate if SET-17 localization affects the chromatin state of primary spermatocytes, we determined the distribution of the presumptive products of the lysine methyltransferase activity of SET-17. SET-17 purified from *E*. *coli* can methylate lysine 4 of histone 3 to the mono- and di-methyl states (H3K4me1 and H3K4me2) but not the tri-methyl state *in vitro* [26]. By immunostaining dissected male germlines, we found that H3K4me1 and H3K4me2 were distributed throughout the nuclei of primary spermatocytes. While both H3K4me1 and H3K4me2 were detectable at SET-17 foci, they were not enriched at SET-17 foci (Figs 5C, 5D, S5E and S5F). These results show that H3K4me1 and H3K4me2 are both present at SET-17 foci and suggest that SET-17 is not the only KMT that can catalyze the generation of H3K4me1 and H3K4me2 in primary spermatocytes.

### SET-17 promotes expression of spermatocyte-specific transcripts

**Fig 6:**
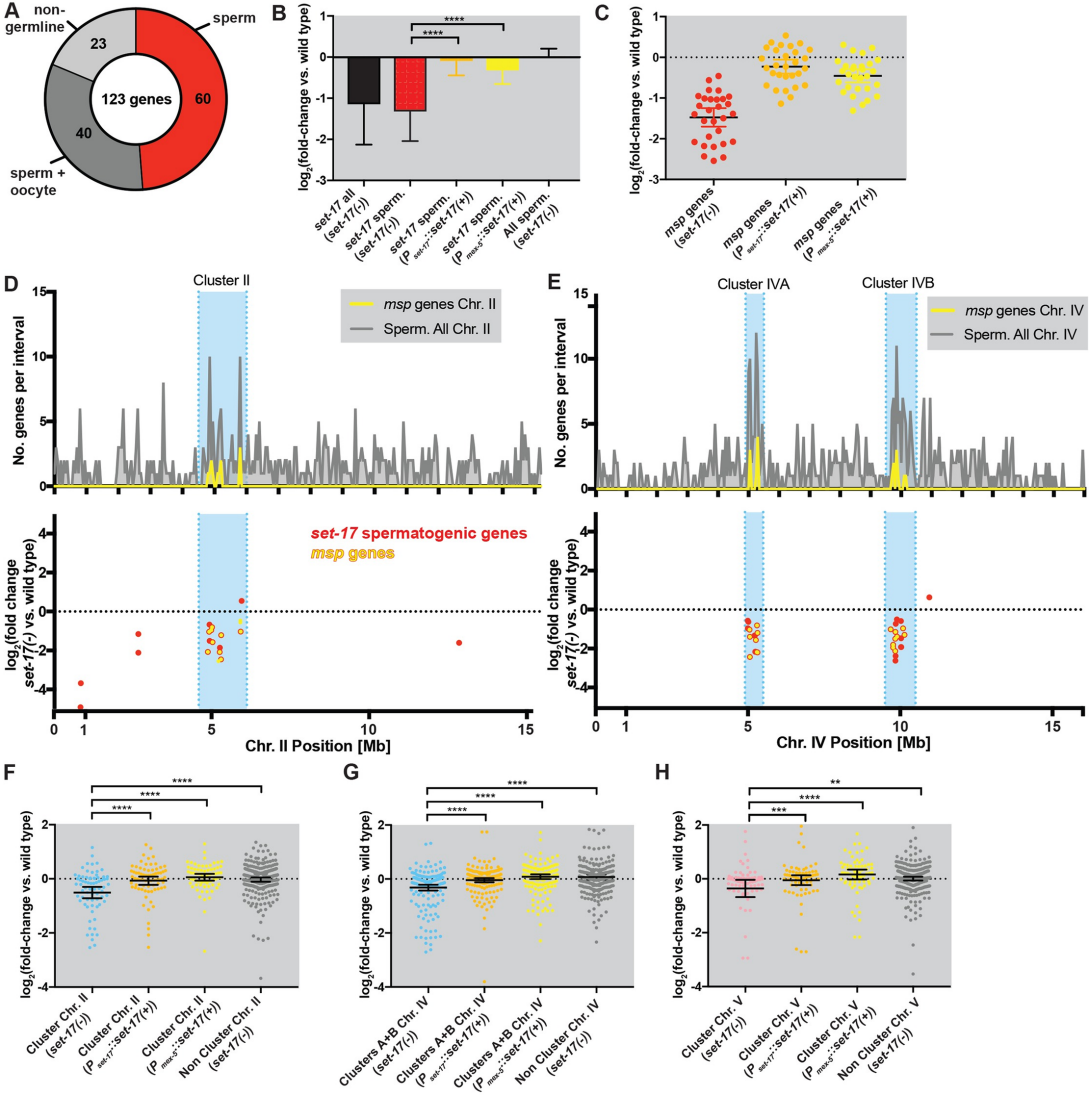
*set-17* promotes expression of *msp* genes in spermatogenic gene clusters. A) Expression categories defined by Ortiz et al. (2014) of 123 transcripts misregulated in *set-17* mutants based on RNAseq studies of whole L4 hermaphrodites. Sperm, enriched in sperm-producing germlines (60, red); sperm + oocyte, genes expressed similarly in sperm and oocytes (40, dark grey); non-germline, genes not detected in dissected germlines (23, light grey). We did not detect genes enriched in oocytes but not sperm. B) *set-17* promotes expression of a subset of spermatogenic transcripts. Average log_2_-fold change in expression vs. the wild-type for all 123 *set-17* misregulated transcripts (*set-17* all) in *set-17* mutants; for the 60 *set-17* misregulated spermatogenic transcripts (*set-17* sperm.) in *set-17* mutants, in *set-17; P*_*set-17*_::*set-17(+)* and in *set-17; P*_*mex-5*_::*set-17(+)* mutants, respectively; and for all 2,306 previously identified spermatogenic transcripts (All sperm.) in *set-17* mutants. **** P < 0.0001, MW exact or KS test. C) *set-17* promotes *msp* expression in the germline. log_2_-fold change in expression vs. the wild type for all 28 *msp* gene transcripts (*msp* genes) in *set-17*, *set-17; P*_*set-17*_::*set-17(+)* and *set-17; P*_*mex-5*_::*set-17(+)* mutants. **** P < 0.0001, MW exact or KS test. D), E) *set-17* spermatogenic genes cluster in the genome. Positional distributions of spermatogenic genes and *msp* genes on chromosomes II and IV and spermatogenic *set-17-*misregulated genes on chromosomes II and IV. Above: histograms of spermatogenic genes on chromosomes (D) II and (E) IV. Blue, spermatogenic gene clusters. Below: log_2_-fold change in expression in *set-17* vs. the wild type from RNAseq studies in whole L4 hermaphrodites for the *set-17* spermatogenic genes (red) on chromosomes (D) II and (E) IV. Yellow, *msp* genes. F) *set-17* promotes expression of spermatogenic genes in a cluster on chromosome *II*. log_2_-fold change in expression vs. wild type from RNAseq studies of whole L4 hermaphrodites for all spermatogenic genes in the spermatogenic gene cluster on chromosome II (blue region in 5D) in *set-17* (blue), *set-17; P*_*set-17*_::*set-17(+)* (orange) and *set-17; P*_*mex-5*_::*set-17(+)* (yellow) as well as for all spermatogenic genes outside of the blue region on chromosome II (grey). Mean +\-95% CI; **** P < 0.0001, MW exact or KS test. G) *set-17* promotes expression of spermatogenic genes in clusters on chromosome *IV*. log_2_-fold change in expression vs. the wild type from RNAseq studies of whole L4 hermaphrodites for all spermatogenic genes in the spermatogenic gene clusters on chromosome IV (blue regions in 5E) in *set-17* (blue), *set-17; P*_*set-17*_::*set-17(+)* (orange) and *set-17; P*_*mex-5*_::*set-17(+)* (yellow), as well as for all spermatogenic genes outside of the blue region on chromosome IV (grey). Mean +\-95% CI; **** P < 0.0001, MW exact or KS test. H) *set-17* promotes expression of spermatogenic genes in a cluster on chromosome *V*. log_2_-fold change in expression vs. the wild type from RNAseq studies of whole L4 hermaphrodites for all spermatogenic genes in the newly identified spermatogenic gene cluster on chromosome V (blue region in S5K) in *set-17* (pink), *set-17; P*_*set-17*_::*set-17(+)* (orange) and *set-17; P*_*mex-5*_::*set-17(+)* (yellow) as well as for all spermatogenic genes outside of the blue region on chromosome V (grey). Mean +\-95% CI shown. Cl. V (*set-17(-)*) vs. Non-Cl. V (*set-17(-)*), ** P < 0.005, MW exact; Cl. V (*set-17(-)*) vs. Cl. V (*P*_*set-17*_::*set-17(+)*), *** P < 0.0005, Wilcoxon test; Cl. V (*set-17(-)*) vs. Cl. V (*P*_*mex-5*_::*set-17(+)*), **** P < 0.0001, Wilcoxon test.

The localization of SET-17 to chromatin in primary spermatocytes coupled with the reduction in *set-17* mutants of FB-MOs, spermatids and fertility suggested that SET-17 functions to promote transcription in primary spermatocytes. To determine the role of *set-17* in spermatocyte gene expression, we analyzed the transcriptome of hermaphrodites at the sperm-producing L4 stage. Loss of *set-17* affected the expression of 123 transcripts, down-regulating them 2-fold on average; this transcriptional misregulation was rescued by wild-type *set-17* expression using the *set-17* promoter (S6A and S6B Fig). We asked if *set-17* affects the expression of genes enriched in spermatocytes. Based on known gene-expression data from individual dissected germlines in the oogenic or spermatogenic state [27], we categorized transcripts into four categories: spermatogenic, oogenic, spermatogenic and oogenic or non-germline. Of the 123 transcripts misregulated in *set-17* mutants (defined as “all *set-17*”), 60 were spermatogenic (“*set-17* spermatogenic”; Fig 6A). The 60 *set-17* spermatogenic transcripts were on average 2-fold downregulated in *set-17* mutants (Fig 6B). Wild-type *set-17* expression in the germline rescued the expression of *set-17* spermatogenic transcripts (Fig 6B). Loss of *set-17* did not affect spermatogenic gene expression globally, as on average the 2306 previously identified spermatogenic transcripts were unchanged in *set-17* (Fig 6B). A rank-correlation analysis of all *set-17* misregulated genes indicated that the most highly misregulated transcripts were enriched for spermatogenic genes (S6C Fig). We conclude that SET-17 functions in the germline to promote the expression of a small subset of spermatogenic genes.

### SET-17 promotes the expression of *msp* genes

Since FB-MO production was reduced in *set-17* spermatocytes and FB-MOs store MSP paracrystals, we asked if the genes encoding MSPs were among the down-regulated spermatogenic transcripts in *set-17* mutants. The *msp* gene family comprises 28 distinct genes that encode identical MSP peptides. While the coding mRNA sequences of the 28 *msp* genes are 95% identical, it is possible to assay the expression level of each *msp* gene individually using RNAseq because of their completely divergent 3’ and 5’ UTRs as well as the randomly distributed single nucleotide polymorphisms that occur throughout the coding sequences. We designed a set of 16 smFISH probes that covered the full length of the *msp* coding sequence. Each individual *msp* gene can be recognized by at least 10 probes, so collectively these probes detect all 28 *msp* transcripts. Collectively the 28 *msp* genes were down-regulated in *set-17* mutants. 26 of 28 *msp* genes were among the 60 *set-17* spermatogenic transcripts (Fig 6C). *msp* expression was reduced to 50% of wild-type levels in *set-17* mutants, and this defect was rescued by the expression of wild-type *set-17* specifically in the germline (Fig 6C). We conclude that *set-17* promotes the expression of *msp* genes.

### SET-17 promotes expression of four clusters of spermatocyte-specific genes

To explore how *set-17* affects the expression of *msp* and other spermatogenic genes, we investigated the genomic positions of the genes encoding the *set-17* spermatogenic transcripts. *msp* genes cluster in three distinct regions in the genome: one on chromosome *II* and two on chromosome *IV* [28]. Non-*msp* spermatocyte-specifically expressed genes are also enriched in these clusters [29]. First, we analyzed the distribution of all known spermatogenic genes on chromosomes *II* and *IV* to confirm the clustering of spermatogenic genes at the *msp* gene loci (blue regions, Fig 6D and 6E). We then plotted the fold-change in expression levels in *set-17* vs. wild-type as a function of genomic position for the *set-17* spermatogenic genes (shown in red; yellow indicates *msp* gene). We found that 53 of the 60 *set-17* spermatogenic genes were located in the spermatogenic gene clusters on chromosomes *II* and *IV* (26 *msp* genes, 27 non-*msp* genes; Fig 6D and 6E). We conclude that *set-17* preferentially promotes expression of genes in spermatogenic gene clusters.

To investigate whether *set-17* might also regulate spermatogenic genes in the clusters on chromosomes *II* and *IV* in addition to the ones already identified in our global analysis of gene expression in *set-17* mutants, we asked if the spermatogenic genes in the clusters were more likely to be down-regulated in *set-17* mutants when compared to spermatogenic genes outside of the clusters. This analysis showed that loss of *set-17* preferentially caused the down-regulation of spermatogenic genes located in the clusters on chromosomes *II* and *IV* and that expression of wild-type *set-17* in the germline rescued this effect (Figs 6F, 6G, S6G and S6H). This preferential down-regulation of spermatogenic genes in clusters remained statistically significant after removing the *msp* genes from the analysis (S6I and S6J Fig).

Many additional spermatogenic genes have been identified since the spermatogenic gene clusters on chromosomes *II* and *IV* were identified. We therefore hypothesized that previously unidentified non-*msp* spermatogenic gene clusters might exist in the *C*. *elegans* genome and that *set-17* might affect the expression of the genes in such a cluster. We therefore analyzed the distribution of spermatocyte-specifically expressed genes defined by Ortiz et al. on all *C*. *elegans* chromosomes [27]. We identified a previously unknown cluster on chromosome *V* (S6K Fig). We analyzed the effect of loss of *set-17* on spermatogenic genes in the chromosome *V* cluster compared to the spermatogenic genes outside the cluster on chromosome *V*. We found that loss of *set-17* specifically reduced expression of the chromosome *V* cluster genes and that this reduction was rescued by expression of wild-type *set-17* specifically in the germline (Fig 6H). These results show that SET-17 acts in the germline to promote expression from four spermatogenic gene clusters on chromosomes *II*, *IV* and *V*.

### SET-17 promotes transcription of *msp* gene clusters in spermatocytes

**Fig 7:**
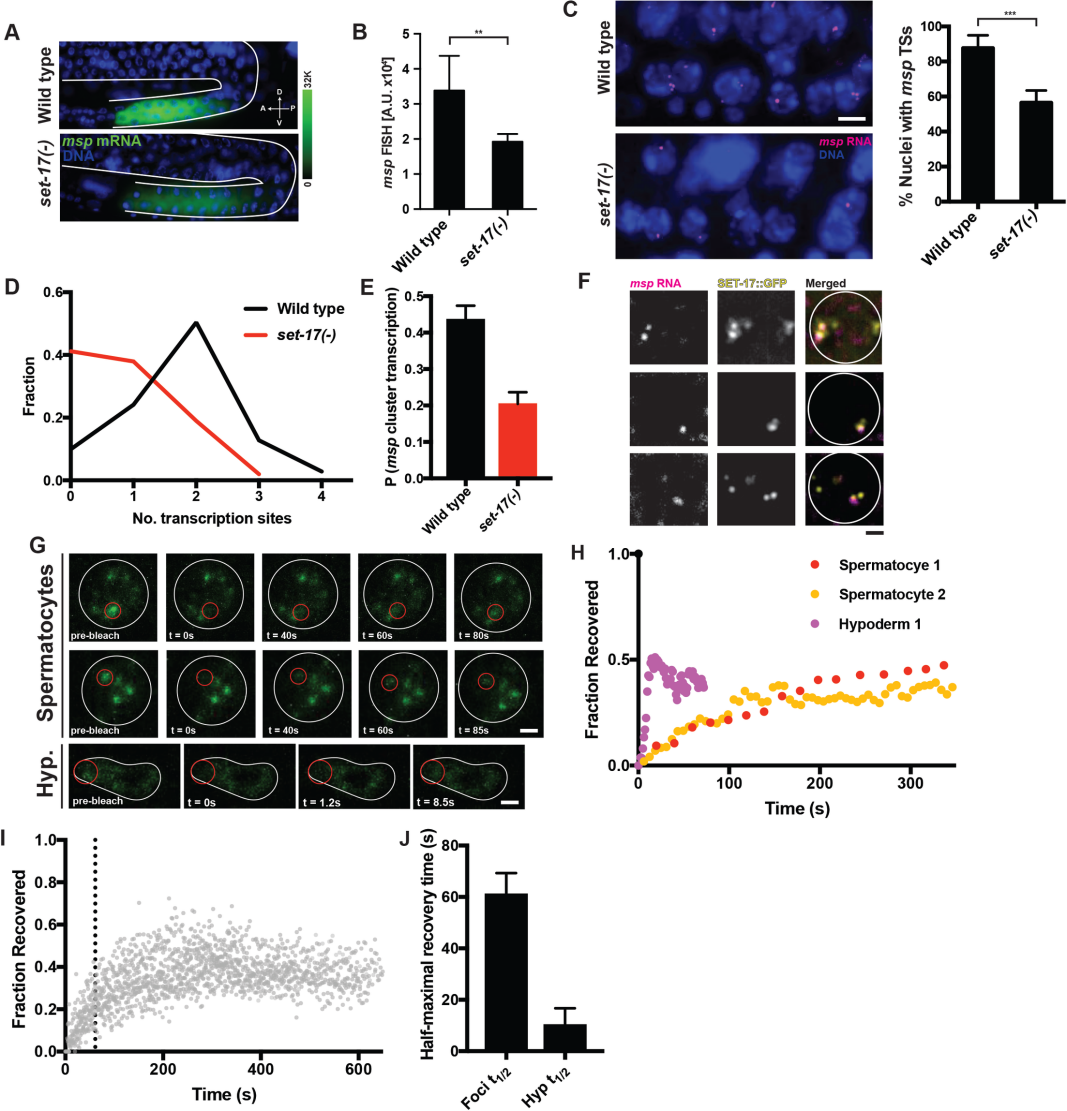
SET-17 promotes transcription at spermatogenic gene clusters. A) Endogenous *msp* mRNA expression determined by smFISH using a probe-set that recognizes transcripts from 28 *msp* genes in wild-type or *set-17* sperm-producing germlines of L4 hermaphrodites. White lines delineate the gonad. Scale bar, 10 μm. B) Levels of endogenous *msp* mRNA as measured by smFISH in mature primary spermatocytes in wild-type and *set-17* L4 hermaphrodites. n > 10, P < 0.001, t-test. C) Left, endogenous *msp* RNA transcription sites (TSs) in the nuclei of wild-type and *set-17* primary spermatocytes in L4 hermaphrodites; scale bar, 5 μm. Right, percent of primary spermatocyte nuclei with at least one *msp* TS (positive). Wild type n = 177, *set-17* n = 199. *** P < 0.0001, t-test. D) Frequency distribution of TSs in primary spermatocyte nuclei of wild-type or *set-17(-)* sperm-producing L4 hermaphrodites. Avg. TSs per nucleus: wild type, 1.75, n = 141; *set-17*, 0.82, n = 153; P < 0.0001, KS or MW test. E) Probability that a single *msp* cluster is transcribed in a wild-type or *set-17* L4 hermaphrodite. Calculated from the TS distributions for wild-type and *set-17* animals shown in (D). P < 0.0001, t-test. F) Co-localization of endogenous *msp* RNA TSs (as visualized by smFISH) and SET-17::GFP (as visualized by GFP protein fluorescence) in the nuclei of primary spermatocytes in the male germline. White circles, nuclear circumference; scale bar, 1 μm. G) Individual frames from an *in vivo* FRAP time series study of two primary spermatocytes and a hypodermal nucleus. White lines, nuclear circumference; scale bar, 1 μm; red circle, focal area of laser bleaching and region of measurement. H) Normalized fluorescence intensity measurement in the area of bleaching for the three experiments shown in (G). t = 0, pre-bleaching intensity. Recovery of SET-17::GFP is 10-fold slower in spermatocyte foci than in hypodermal nuclei. I) Normalized FRAP data for 16 experiments examining spermatocyte foci. Black dotted line indicates average half-maximal recovery time, 61 s, calculated from exponential fits for each experiment. J) Average half-maximal recovery times from FRAP studies of SET-17::GFP in spermatocyte foci (Foci t_1/2_) or hypodermal nuclei (Hyp t_1/2_). Error bars: SEM; P < 0.05, t-test.

That SET-17 promotes the expression of spermatogenic genes suggests that SET-17 promotes their transcription in spermatocytes. Using the set of *msp* smFISH probes described above, we found that in mature primary spermatocytes of L4 hermaphrodites loss of *set-17* caused a 50% reduction of total *msp* mRNA (Fig 7A and 7B). We conclude that *set-17* is needed for full *msp* expression in spermatocytes.

To determine the transcriptional activity of *msp* genes, we quantified *msp* RNA foci at transcription sites. RNA polymerase II transcription occurs in bursts of several transcripts, causing the transient accumulation of pre-mRNAs at transcription sites [30]. The transcriptional activity of a gene transcribed by RNA polymerase II can be determined by quantifying nuclear RNA foci by smFISH [31,32]. We examined primary spermatocytes in L4 hermaphrodites and counted *msp* transcription sites in the nuclei of primary spermatocytes. We detected transcription sites in most wild-type spermatocyte nuclei and found that *msp* transcription sites were reduced in number in the spermatocytes of *set-17* mutants (Fig 7C and 7D). We scored primary spermatocyte nuclei as positive or negative for *msp* transcriptional activity, depending on whether at least one *msp* transcription site was detectable. The percentage of positive nuclei was reduced in *set-17* primary spermatocytes to about 60% of that of wild-type (Fig 7C), suggesting that *msp* gene transcription is reduced in *set-17* spermatocytes. To determine the transcriptional activity of single *msp* gene clusters, we counted the number of transcription sites per nucleus in wild-type and *set-17* L4 hermaphrodite spermatocytes (Fig 7D). The median number of transcription sites per primary spermatocyte nucleus was two in wild-type and one in *set-17* animals. We computed the probability of individual *msp* gene cluster transcription from the observed distribution of transcription sites (S7A and S7B Fig, Methods). Strikingly, we found that loss of *set-17* caused a 50% reduction in the probability of transcriptional activity of individual *msp* clusters (Fig 7E). These results demonstrate that *set-17* regulates the transcriptional activity of *msp* genes in primary spermatocytes.

### *msp* transcription sites co-localize with SET-17 foci

SET-17 is required for the transcription of *msp* gene clusters, and SET-17 localizes to foci in spermatocyte nuclei (Fig 5B). We hypothesized that SET-17 foci might localize to *msp* gene clusters to promote their transcription. Strikingly, we found that *msp* transcription sites colocalized with SET-17::GFP foci in primary spermatocytes (Fig 7F). 90% of *msp* transcription sites occurred at SET-17::GFP foci. *msp* transcription sites occurred in the absence of SET-17::GFP foci only in very early primary spermatocytes with either one or no SET-17::GFP foci. The colocalization of SET-17::GFP foci with *msp* transcription sites is consistent with the hypothesis that SET-17 functions to promote but might not be absolutely essential for *msp* gene expression.

### SET-17 foci are stable nuclear structures with slow diffusion and liquid-like properties

To characterize structural and dynamic features of SET-17 foci, we used time-lapse imaging and fluorescence recovery after photo-bleaching (FRAP) studies of individual SET-17::GFP foci in spermatocytes *in vivo*. We found that individual SET-17::GFP foci persisted in immobilized 1-day adult males for the duration of our experiments (~10 min) and that SET-17::GFP fluorescence recovered after photo-bleaching in individual foci (Fig 7G). By quantifying the SET-17::GFP fluorescence recovery for individual foci we determined the rate of exchange of SET-17::GFP in the foci (Fig 7H). SET-17::GFP recovered fluorescence in spermatocyte foci on a time-scale of tens of seconds (Fig 7H), while recovery occurred on a time scale of seconds in hypodermal nuclei, where the protein is distributed throughout the nucleus (Fig 7G and 7H). The time of half-maximal fluorescence recovery is a measure of the equilibrium constant of the exchange of SET-17 from the foci with the environment. The time of half-maximal recovery of SET-17::GFP in spermatocyte foci was significantly slower than that in hypodermal nuclei (Fig 7I and 7J). These data revealed a barrier to SET-17 exchange from the foci with the nucleoplasm in spermatocytes. Foci with diffusion barriers can be the consequence of liquid-liquid phase separation [33]. In addition to slow diffusion, liquid-liquid phase-separated droplets exhibit fusion and fission, which can distinguish them from other macro-molecular assemblies with slow diffusion rates [33]. Although SET-17 foci were largely stationary in immobilized animals, we did observe some fusion and fission of SET-17 foci (S7C and S7D Fig). We speculate that SET-17 foci might be novel phase-separated stable nuclear structures associated with *msp* gene clusters that facilitate *msp* transcription.

### ELT-1 promotes *msp* expression and fertility together with SET-17

**Fig 8:**
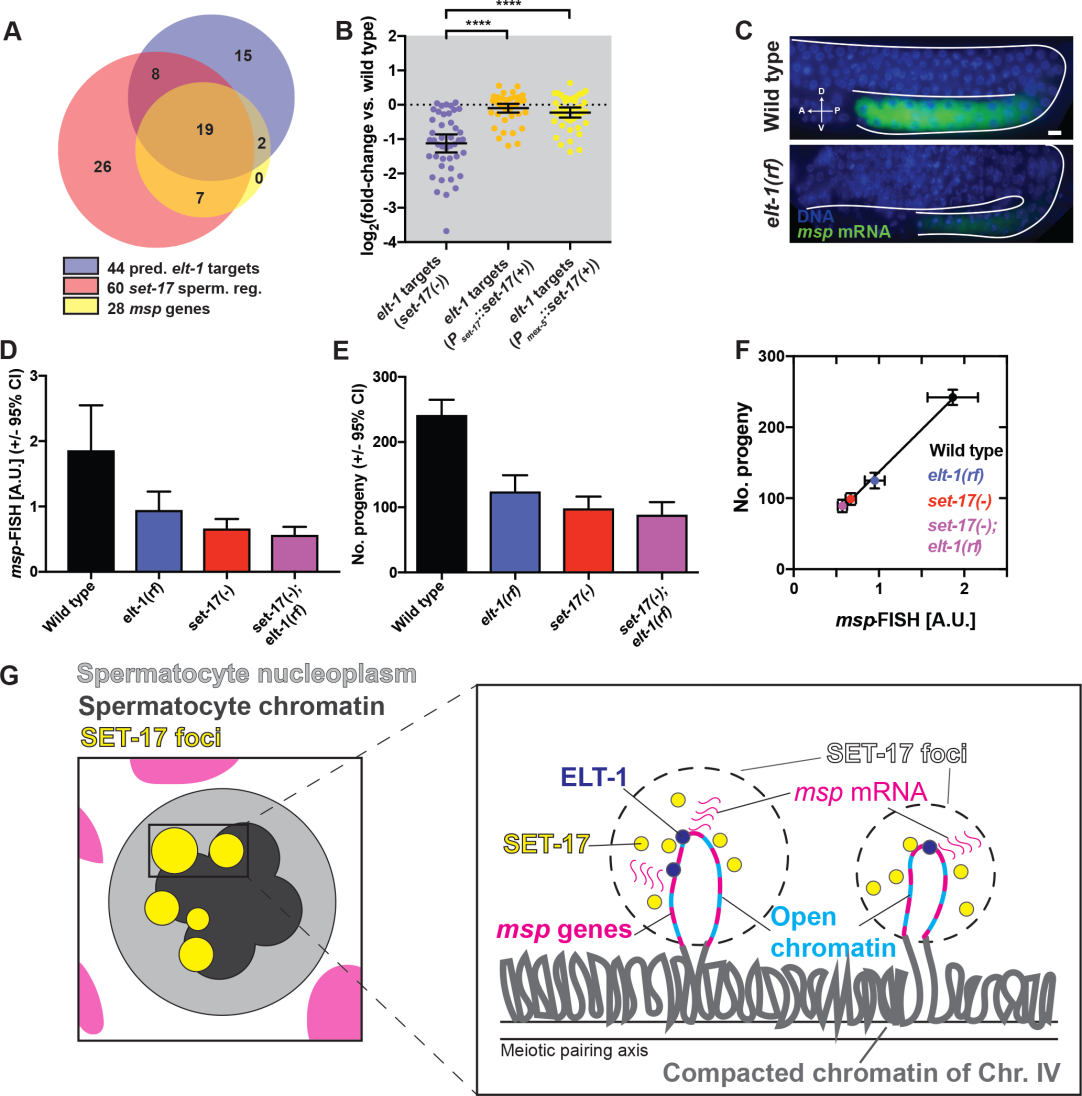
ELT-1 acts with SET-17 to promote spermatogenic gene cluster expression and fertility. A) Overlays among the sets of the 44 predicted spermatogenic *elt-1* targets (from data of del Castilles-Olivera et al., 2009), the 60 spermatogenic *set-17* misregulated genes (from our Fig 6A) and the 28 *msp* genes. B) Average log_2_-fold change in expression from RNAseq studies compared to the wild type for the 44 predicted *elt-1* target genes in *set-17* (purple), *set-17; P*_*set-17*_::*set-17(+)* (orange) and *set-17; P*_*mex-5*_::*set-17(+)* (yellow) mutant whole L4 hermaphrodite. **** P < 0.0001, MW or KS-test. C) Endogenous *msp* mRNA detection by smFISH in primary spermatocytes of *elt-1* partial loss-of-function (rf) L4 hermaphrodites. Scale bar, 10 μm. D) Endogenous *msp* mRNA levels as visualized by smFISH in mature primary spermatocytes in wild-type (black), *elt-1* (purple), *set-17* (red) and *set-17; elt-1* (magenta) L4 hermaphrodites. n > 15. E) Number of progeny of wild-type (black), *elt-1* (purple), *set-17* (red) and *set-17; elt-1* mutants (magenta). n > 20. F) Fertility correlates with endogenous *msp* mRNA levels as visualized by smFISH (data from Fig 7C and 7D). Pearson-correlation r = 0.9981, P < 0.002 (2-tailed). G) Model of SET-17 foci composition and function in *msp* gene expression in primary spermatocyte nuclei at spermatogenic gene clusters. See text for details.

Our data suggest that the reduction of fertility in *set-17* mutants is caused by a reduction of transcription of *msp* genes. The transcriptional regulator ELT-1 can bind *in vitro* to a promoter motif found upstream of 44 spermatocyte-specifically expressed genes, including 19 *msp* genes [34]. We showed that these 44 predicted *elt-1* target genes were enriched in the three known spermatogenic clusters on chromosomes *II* and *IV* (33 out of 44, S8A and S8B Fig) and that 26 are also among the *set-17* spermatogenic genes (Fig 8A). All 44 predicted *elt-1* targets were downregulated in *set-17* mutants, and their expression was restored by expression of wild-type *set-17* in the germline (Fig 8B). Thus SET-17 and ELT-1 might function together in *msp* transcription.

Does ELT-1 function in endogenous *msp* regulation and fertility? Because *elt-1* is required for viability, we examined a viable partial loss-of-function mutant of *elt-1*. *elt-1(ku419)* carries a P298S missense mutation in one of the two DNA-binding domains of ELT-1 [35]. Using smFISH, we found that this *elt-1* mutation caused a ~50% reduction in endogenous *msp* transcript levels in primary spermatocytes, similar to the reduction in *set-17* mutants (Fig 8C and 8D). *msp* mRNA levels of *set-17; elt-1* double mutants were indistinguishable from those of *set-17* single mutants (Fig 8D). These results indicate that ELT-1 regulates *msp* expression together with rather than independently of SET-17.

*elt-1* mutants were reduced in fertility to ~50% of that of the wild-type, similar to *set-17* mutants (Fig 8E). The *elt-1* mutation did not enhance the fertility defect caused by a *set-17* mutation, indicating that *elt-1* and *set-17* act together to control fertility. We conclude that ELT-1 and SET-17 function together to promote *msp* expression and fertility.

To ask how fertility and *msp* expression might be related, we plotted fertility as a function of *msp* expression levels for wild-type, *set-17*, *elt-1*, *set-17; elt-1*. *msp* mRNA levels were tightly correlated with fertility (Fig 8F, r = 0.9981, P < 0.0001), suggesting that *msp* expression levels might determine fertility. We explored this relationship further by plotting *msp* expression and fertility for all strains, including the *msp* expression data from the RNAseq experiments (S8C Fig, R^2^ = 0.97). *msp* expression and fertility corresponded for *set-17* and *elt-1* mutants, as well as for the *set-17(+)* rescued strains, independent of experimental measurement, further demonstrating a correlation between *msp* expression and fertility.

We conclude that SET-17 functions in fertility by promoting the transcription of *msp* gene clusters.

## Discussion

Our studies have defined the first comprehensive tissue-specific expression map of KMTs in any organism. We discovered that SET-17, the only *C*. *elegans* member of the PRDM9/7 SET-domain family, promotes sperm production and fertility in primary spermatocytes. SET-17 localized to chromatin-associated nuclear foci (Fig 8G) and was required for the transcription of spermatocyte-specific genes, including the *msp* genes. Levels of *msp* gene expression in primary spermatocytes, spermatid number and fertility correlated. The existence of clusters of spermatocyte-specific genes in the *C*. *elegans* genome [28,29] and our findings concerning cluster gene co-expression and transcriptional co-regulation by SET-17 suggest that gene clustering is important in sperm production and male fertility.

### Implications for the evolutionary origin and function of mammalian PRDM9 and PRDM7

*set-17* is one of only two PRDM-type KMTs in *C*. *elegans*. The SET-17 protein is a member of the PRDM9/7 SET-domain family but lacks any additional domains, such as the KRAB domain of PRDM9 and PRDM7 or the Zn fingers of PRDM9 (S2B Fig) [11]. In the mammalian germline, PRDM9 is involved in the positioning of DNA double-strand breaks for recombination [11,36,37]. Loss of PRDM9 function causes infertility in male mice as sperm arrest in meiotic prophase [38]. It is unknown how PRDM9 recruits to its target loci the recombination machinery for double-strand break induction. We speculate that PRDM9 might function in SET-17-foci-like nuclear bodies for the initiation of double-strand breaks. Indeed, recent analysis of the induction of double-strand breaks and meiotic recombination have revealed protein accumulation in distinct foci and led to the proposal that such structures represent “recombinosomes” [39]. Molecular analysis of PRDM9 transcripts in whole mouse testes identified a short isoform that lacks the Zn-Finger array and the function of which is unknown [38]. Perhaps this short SET-domain-only isoform of mammalian PRDM9 functions in transcriptional regulation in mammalian spermatocyte development, analogous to SET-17 function in *C*. *elegans* spermatocytes. The function of PRDM7 remains unexplored.

### A SET-17-like transcriptional regulator might act in mammalian sperm production

Loss of the human H3K4me2 demethylase LSD1 in the paternal germline can cause developmental defects in offspring [40,41]. The *C*. *elegans* ortholog of LSD1, SPR-5, functions in transgenerational germline immortality [42]. Mutation of *set-17* can suppress the progressive sterility and accumulation of H3K4me2 in *spr-5* mutants [26]. Intriguingly, transcriptional changes associated with loss of fertility in *spr-5* mutants are enriched for *set-17* spermatogenic genes, including a subset of the *msp* genes (S8E Fig, P < 1E-20). Many of the spermatocyte-specific genes that are down-regulated in *set-17* are up-regulated in late-generation adult *spr-5* mutant hermaphrodites. Based on these observations, we suggest that *set-17* promotes spermatogenic gene expression from clusters and *spr-5* restricts this activity and that PRMD9 or another SET-17-like chromatin regulator functions in mammalian sperm production to deposit chromatin marks that must be appropriately erased by LSD1 for proper development.

### *msp* locus bodies are analogous structures to histone locus bodies

Histone genes cluster in the genome, are highly expressed and associate with distinct nuclear structures, known as histone locus bodies (HLBs), to facilitate their expression [43]. Replication-dependent histone genes are transcribed during S-phase to produce histones for the replicated genome. Multiple histone genes in each cluster encode the same histone protein, analogous to *msp* genes in *C*. *elegans*. Like histone genes, *msp* genes are short (~400 bp) and lack introns. We propose to call the SET-17 foci at *msp* gene clusters *msp* locus bodies (MLBs) by analogy with HLBs.

Like HLBs, MLBs are stable over long periods of time, exhibit slow exchange of protein components and localize to transcriptionally active genomic loci. We also observed instances of MLB fusion and fission (S7 Fig). Dynamic spatial clustering of RNA polymerase II at loci of active transcription has been observed in mouse embryonic fibroblasts [44]. MLBs might function to facilitate the clustering of RNA polymerase II at *msp* gene clusters in *C*. *elegans* spermatocytes. We suggest that spatially restricted nuclear structures concentrate chromatin factors and transcription machinery to support the controlled activation of transcription.

### Spatial restriction of chromatin factors contributes to tissue-specific regulation of transcription

That the expression of most putative KMTs is restricted to only one or two postembryonic tissues suggests that KMTs function broadly in tissue-specific chromatin regulation. Further analysis of KMT specialization, localization and redundant function should enable the identification of additional aspects of chromatin regulation and have important implications for the understanding of chromatin biology in the context of development and disease. For example, a view of oncogenesis is emerging in which tumors originate as a consequence of cell-fate transformations in the cell-type of tumor origin [18]. One focus has been identifying master transcription factors or chromatin drivers of tissue-specific gene expression that might be involved in these cell-fate transformations. Our data show that SET-17 is a novel type of chromatin-modifying driver of tissue-specific transcription states. We suggest that SET-17-like tissue-specific chromatin modifiers are important for transcriptional regulation in development and disease.

## Materials and methods

For complete material and methods, see Supporting Information.

### Single-molecule fluorescence *in situ* hybridization (smFISH)

Probe design and smFISH experiments were performed essentially as described elsewhere [19] with 10% formamide in the hybridization buffer. Samples were incubated overnight at 30˚C and washed for 30 min twice. Imaging was performed using an inverted microscope with a 100x oil objective (Nikon, NA 1.4). smFISH signal was detected using a Pixis 1024 (Princeton Instruments) with exposure times of 2 sec. For *msp* FISH the high fluorescence intensity required exposure times of 500 msec. and a scMos Orca Flash 4.0 camera (Hamamatsu) was used for fluorescence detection.

### Broodsize measurements

For broodsize determination, young L4 animals were individually placed on plates with food and transferred to fresh plates every 24 hr for 3 days. The number of progeny was determined by counting the number of adult animals produced over 6 days. All experiments were performed at 25˚C. Strains were maintained at 20˚C and transferred to 25˚C.

## Supporting information

S1 File (PDF)

S2 File (XLSX)

S1 Fig SET-domain alignment of human and *C*. *elegans* lysine methyltransferases and *set-17* and *set-2* mRNA detection by smFISH studies of whole *C*. *elegans*. A) Similarity tree based on the pairwise alignment scores of all *H*. *sapiens* and *C*. *elegans* SET-domains. B) Representative images of smFISH labeling of *set-17* and *set-2* mRNAs, respectively, in the germline of a wild-type adult hermaphrodite. Maximum projection of 45 Z-slices. Scale bar, 20 μm. The grey area covers an image processing artifact.
(TIF)

S2 Fig Summary of endogenous KMT mRNAs detected in the germline of L1 and wild-type adult hermaphrodites. Blue, expressed in primordial germ cells; purple, expressed throughout the adult hermaphrodite germline; yellow, expressed specifically in oocytes in the adult hermaphrodite germline, but not in the primordial germ cells.
(TIF)

S3 Fig Gene structure, mutations and evolutionary conservation of SET-17. A) Above: Gene model of *set-17* with the SET-domain and mutations used indicated. Coding sequence, black; SET-domain, red; thin lines, introns. Scale bar, 100 bp. Below: Protein domain structures of *H*. *sapiens* PRDM9 and *C*. *elegans* SET-17. Amino acid percent identity determined by ClustalW alignment. N, N-terminus; C, C-terminus. B) Full-length protein domain structure of SET-domain proteins from *C*. *elegans* and *H*. *sapiens*, ordered by similarity of their SET-domains and germline expression in the respective organism is indicated. Red: PRDM9/7-family SET-domain; Green: PRDM1-family SET-domain; Orange: Clr4-family SET-domain. The alignment distances are qualitative. Germline expression of PRDM7 was detected by sequencing of whole human tissues and PRDM7 expression is enriched in testes (GTEx portal, Illumina Body Map). SET-17 is more similar to PRDM7 and PRDM9 than to any of the other SET-domain proteins. *C*. *elegans* BLMP-1 is the ortholog of *H*. *sapiens* PRDM1 and neither is expressed in the germline. SET-17 and BLMP-1 are the only PRDM-family SET-domains in *C*. *elegans* (see S1A Fig). The SET-domain of *C*. *elegans* SET-11 is least similar to the PRDM-type SET-domains. SET-domain identity between PRDM9 and SET-17: 48%; SET-domain identity between PRDM7 and SET-17: 47%; SET-domain identity between PRDM9 and PRDM7 97%; BLMP-1 and PRDM1: 41%; SET-17 and PRDM1: 38%; SET-17 and BLMP-1: 30%; SET-11 and PRDM9: 25%; SET-11 and PRDM1: 20%; SET-11 and SET-17: 23%; SET-11 and BLMP-1: 15%. (% identity obtained with ClustalW, SET-domain sequences from Uniprot). C) Broodsizes of *set-17* and *set-17* double mutants with select germline-expressed KMTs. n > 15.
(TIF)

S4 Fig *set-17* promotes the number and size of major sperm protein vesicles in primary spermatocytes. A) Number of FB-MOs per primary spermatocyte in wild type, *set-17* and *set-17; P*_*set-17*_::*set-17(+)* adult male, corrected for mean FB-MO size and cross-sectional area. n = 10; * P < 0.05, t-test. B) Quantification of special membrane structures (SMS) in mature spermatids of wild-type, *set-17* and *set-17; P*_*set-17*_::*set-17(+)* adult males. Arrowheads, representative SMS. n > 18. Scale bar, 500 nm.
(TIF)

S5 Fig SET-17 is expressed in the nuclei of primary spermatocytes and enriched in chromatin-associated foci. A) Numbers of SET-17::GFP foci per spermatocyte nucleus in early, mid and late primary spermatocytes in adult males. Karyo = karyosome, nuclei that are undergoing transformation to secondary spermatocytes. B) Confocal image of SET-17::GFP in spermatocytes of a live immobilized L4 hermaphrodite. Representative Z-section. Scale bar, 5 μm. C) Confocal image of SET-17::GFP in the oocyte-producing germline of a live immobilized adult hermaphrodite. Representative Z-section. Scale bar, 5 μm. D) Confocal image of SET-17::GFP in the hypoderm of a live immobilized adult hermaphrodite. Representative Z-section. Scale bar, 5 μm. E) & F) Immunostaining of primary spermatocyte nuclei from an adult male expressing SET-17::GFP as in Fig 4C & 4D. Nuclei are stained for H3K4me1 (E) and H3K4me2 (F), as well as SET-17::GFP. DNA stained by DAPI.
(TIF)

S6 Fig *set-17* promotes expression of *msp* genes in spermatogenic gene clusters. A) Summary of transcriptome analysis of expression in wild-type and *set-17* mutants. logFC: logarithm (base 2) of the fold-change *set-17* / wild-type; logCPM: logarithm (base 2) of the counts per million, a measure of expression level of a transcript. The 123 transcripts that were identified as significantly different between *set-17* and wild-type are indicated in red (see methods, EdgeR). B) Cumulative distribution of the fold-change over wild-type values for the 123 significantly misregulated genes in *set-17*, plotted for *set-17* and the two rescue lines expressing wild-type *set-17* from its endogenous promoter (*set-17; P*_*set-17*_::*set-17(+)*, orange) or a germline-specific promoter (*set-17; P*_*mex-5*_::*set-17(+)*, yellow), compared with all 2306 previously identified spermatogenic transcripts in *set-17* (black). C) Rank-correlation analysis of the spermatogenic gene enrichment in the 123 significantly misregulated transcripts in *set-17* mutants. Plotted here is the cumulative fraction of genes that are spermatogenic for a given rank (the distribution drops for every non-spermatogenic gene in the list of 123 misregulated genes). D) The relative average levels of expression of all 28 *msp* genes for the indicated genotypes, based on the RPKM values of individual *msp* genes. These are the same data as in Fig 5C but depicted as a percentage rather than a log ratio. E) Levels of *msp* expression of all 28 *msp* genes correlate with *set-17* transcript levels as measured by RNAseq in wild-type, *set-17*, *set-17; P*_*set-17*_::*set-17(+)* (orange) and *set-17; P*_*mex-5*_::*set-17(+)* (yellow) L4 hermaphrodites. F) Broodsizes correlate with *set-17* transcript levels as measured by RNAseq in wild-type, *set-17*, *set-17; P*_*set-17*_::*set-17(+)* (orange) and *set-17; P*_*mex-5*_::*set-17(+)* (yellow) L4 hermaphrodites. G) Cumulative distribution of the fold-change vs. wild-type values from RNAseq studies of L4 hermaphrodites for the 72 genes in the spermatogenic gene cluster on chromosome *II* in *set-17* (blue), *set-17; P*_*set-17*_::*set-17(+)* (orange) and *set-17; P*_*mex-5*_::*set-17(+)* (yellow) and for the 365 spermatogenic genes on chromosome *II* not in the cluster in *set-17* (grey). These data are the same as in Fig 5F. These distributions were used to calculate statistical significance using non-parametric tests. H) Cumulative distribution of the fold-change vs. wild-type values from RNAseq studies of L4 hermaphrodites for the 176 genes in the two spermatogenic gene clusters on chromosome *IV* (Cl. IVA+B) in *set-17* (blue), *set-17; P*_*set-17*_::*set-17(+)* (orange) and *set-17; P*_*mex-5*_::*set-17(+)* (yellow) and for the 328 spermatogenic genes on chromosome *IV* not in the cluster in *set-17* (grey). These data are the same as in Fig 5G. These distributions were used to calculate statistical significance using non-parametric tests. I) Cumulative distribution of the fold-change vs. wild-type values from RNAseq studies of L4 hermaphrodites for the 72 genes in the spermatogenic gene cluster on chromosome *II* in *set-17*—minus the 12 *msp* genes (blue) and for the 328 spermatogenic genes on chromosome *IV* not in the cluster in *set-17* (grey). P < 0.0001, MW or KS test. J) Cumulative distribution of the fold-change over wild-type values from RNAseq studies of L4 hermaphrodites for the 176 genes in the two spermatogenic gene clusters on chromosome *IV* (Cl. IVA+B) in *set-17* minus the 16 *msp* genes (blue) and for the 328 spermatogenic genes on chromosome *IV* not in the cluster in *set-17* (grey). P < 0.0001, MW or KS test. K) Histogram of spermatogenic genes on chromosome *V* in 50 kb bins. We identified a previously uncharacterized sperm-gene cluster around position 1.5 x 10^7^ bp. P < 0.0001, hypergeometric test with FDR correction.
(TIF)

S7 Fig SET-17 promotes transcription at spermatogenic gene clusters. A) Empirical transcription site distribution of *msp* genes in primary spermatocytes for wild-type and *set-17* as in Fig 6F. Dotted lines indicate the simulated distribution of TSs generated by a binomial model using the following parameters derived from the empirical data. Number of states, the observed maximum number of states in either the wild type or *set-17* was equal to four; mean, empirical means of the observed distributions of TSs in wild type and *set-17* (respectively, solid lines) normalized by four, the maximum number of states observed. B) Goodness-of-fit analysis of the simulated and the empirical data in S6A Fig showing the simulated data as a function of the empirical data and examining their correlation. Wild-type, R^2^ = 0.853; *set-17*, R^2^ = 0.987. C) Above: frames of a confocal movie showing the fusion of two SET-17::GFP foci in the nucleus of a primary spermatocyte in an immobilized adult male. Scale bar, 1 μm. Below: Quantification of SET-17::GFP foci signal intensity over time, showing the sudden increase in fluorescence of one focus, while another focus stayed constant. D) Above: frames of a confocal movie showing the fission of a SET-17::GFP focus in the nucleus of a primary spermatocyte in an immobilized adult male. Scale bar, 1 μm. Below: Quantification of SET-17::GFP focus signal intensity over time, showing the sudden decrease in fluorescence of the focus.
(TIF)

S8 Fig ELT-1 acts with SET-17 to promote spermatogenic gene cluster expression and fertility. A) Histogram of the positions of the predicted *elt-1* target genes on chromosome *II* in 50 kb bins (purple), plotted with the distribution of positions of all spermatogenic genes (grey) and all *msp* genes (yellow) on chromosome *II*. The spermatogenic gene cluster is indicated in light blue. B) Histogram of the positions of the predicted *elt-1* target genes on chromosome *IV* in 50 kb bins (purple), plotted with the distribution of positions of all spermatogenic genes (grey) and all *msp* genes (yellow) on chromosome *IV*. The spermatogenic gene clusters are indicated in light blue. C) Fertility (No. progeny % wild type) plotted as a function of *msp* expression (average *msp* RPKM or as *msp* FISH % wild type), data combined from S5F Fig and Fig 7F. R^2^ = 0.974. D) Representative confocal images of SET-17::GFP in spermatocytes of a live immobilized L4 hermaphrodite treated with RNAi against *elt-1*, *unc-22* (Ctrl) or *gfp*, respectively. RNAi against *elt-1* did not affect SET-17::GFP foci. E) Overlaps between (1) all 445 genes misexpressed in progressively sterile *spr-5* adult hermaphrodites and the 123 genes misregulated in *set-17* L4 hermaphrodites, (2) all 445 genes misexpressed in progressively sterile *spr-5* adult hermaphrodites and the 60 *set-17* spermatogenic genes misexpressed in L4 hermaphrodites and (3) the 202 spermatogenic genes misexpressed in progressively sterile *spr-5* adult hermaphrodites and the 60 *set-17* spermatogenic genes misexpressed in L4 hermaphrodites. *spr-5* gene expression data from Katz et al. (2009); spermatogenic gene expression categories from Ortiz et al. (2014). P < 1E-20 for each category, hypergeometric test.
(TIF)
