# Supplementary material for: A Caenorhabditis elegans protein with a PRDM9-like SET domain localizes to chromatin-associated foci and promotes spermatocyte gene expression, sperm production and fertility
Source: PLoS Genet. 2018 Apr 27;14(4):e1007295. doi: 10.1371/journal.pgen.1007295 (PMC5942854; doi:10.1371/journal.pgen.1007295)
Supplement: S1 File — (PDF) [file pgen.1007295.s001.pdf]

Supplementary Information for

A *C. elegans* protein with a PRDM9-like SET domain localizes to chromatin-associated foci and promotes spermatocyte gene expression, sperm production and fertility

Authors

Christoph Engert[1,2,3], Rita Droste[1,2], Alexander van Oudenaarden[1,4+] & H. Robert Horvitz[1,2*]

1 – Dept. Biology, MIT, Cambridge, MA 02139

2 – Howard Hughes Medical Institute, MIT, Cambridge, MA 02139

3 – Computational & Systems Biology Graduate Program, MIT, Cambridge, MA 02139

4 – Dept. Physics, MIT, Cambridge, MA 02139

* Corresponding author: horvitz@mit.edu

+ Present address: Hubrecht Institute, 3508 AD Utrecht, NL

Supplemental Materials and Methods

Strains

MT14401	*set-12(n4442) X*
MT14911	*set-4(n4600) II*
RB1383	*set-5(ok1568) V*
MT16425	*set-3(n4948) I*
MT16426	*set-9(n4949) IV*
MT16973	*met-1(n4337) I*
MT17097	*set-22(n5015) V*
MT17453	*set-25(n5021) III*
MT20954	*set-14(n5562) II*
MT21441	*set-8(tm2113) X*
MT21442	*set-28(n4953) X*
MT21444	*met-2(n4256) III*
MT21445	*set-13(n5012) II*
MT21446	*set-6(ok2195) X*
MT21449	*set-29(ok2772) I*
MT21451	*set-17(n5017) II*
MT21453	*set-17(n5017) II; unc-119(ed3) III; cxTi10882 IV*
MT21455	*set-31(ok1482) V*
MT21478	*set-17(n5017) II; unc-119(ed3) III; nSi3[P_{set-17}::set-17(+)::GFP::set-17 3' UTR unc-119(+)] IV*

MT22383	*set-17(n5017) II; unc-119(ed3) III; nSi5[P_{mex-5}::set-17(+)::GFP::set-17 3' UTR unc-119(+)] IV*
MT22787	*set-17(n5017) set-14(n5562) II*
MT22794	*set-29(ok2772) I; set-17(n5017) II*
MT22795	*set-17(n5017) II; set-28(n4953) X*
MT22918	*set-17(n5017) II; fog-2(q71) V*
MT22920	*fog-2(q71) V*
MT22931	*set-32(ok1457) I*
MT23054	*set-17(n5017) II; nSi3 IV; fog-2(q71) V*
MT23056	*set-17(n5017) II; nSi5 IV; fog-2(q71) V*
MH4799	*elt-1(ku419) IV*
MT24115	*set-17(gk417488) II*
MT24119	*set-2(ok952) III*
MT24120	*set-17(n5017) II; set-22(n5015) V*
MT24121	*set-17(n5017) II; set-5(ok1568) V*
MT24122	*set-17(n5017) II; elt-1(ku419) IV*
MT24123	*set-17(n5017) II; set-2(ok952) III*
	set-9(n4949) set-26(tm3526) IV

KMT identification

Using Blast searches, we confirmed the existence of 36 putative SET-domain-containing KMTs encoded by the *C. elegans* genome, as reported by Andersen and Horvitz (2007). We scored SET domain sequence alignments with the BLOSUM62 scoring matrix and used simple bootstrapping to assess statistical robustness (Software package Geneious, Biomatters Ltd.). SET domain orthology relationships were defined as mutual best matches in BlastP searches using only the core SET domain of each protein. We then compared these results with mutual best matches using the entire protein sequence. We refer to genes as orthologs only if they were mutual best matches in both comparisons.

KMT probe sets for smFISH

smFISH probe sets were designed using standard parameters (Raj et al., 2008; 2010): 20 nt oligos at least 2 nt apart from each other with a target GC-content of 45%. We computationally checked all oligos for off-target matches in the *C. elegans* genome and eliminated those with perfect off-target matches. We ordered 48 20 nt oligos for each KMT mRNA. If mRNAs were too short to accommodate 48 probes, we obtained the highest number of high-quality probes possible. Probes and their sequences are available upon request. We ordered the probes as individual 20 nt oligos each with a 3' amine residue. Fluorescent dyes were coupled to the oligos using reactive N-hydroxysuccinimide (NHS) esters in aqueous solution. We coupled all KMT probe sets to Cy5 NHS-mono ester (GE Health Care, PA15101) and Alexafluor 594 NHS-mono ester (Thermo Fisher Scientific, A20004).

smFISH

smFISH studies were performed essentially as described (Raj et al., 2008; 2010; Ji and van Oudenaarden, 2012). Animals were collected in M9, washed 3 times with water and fixed in 4% formaldehyde, 1x PBS for 30-45 min at room temperature. Samples were stored in 70% EtOH at 4˚C until hybridization for at least 24 hrs. For hybridization, animals were prepared in Wash Buffer (10% Formamide, 2x SSC), and hybridization was done in Hybridization Buffer (10% formamide) at 30˚C overnight. Samples were washed in Wash Buffer for 2x 30 min, including 5 ng/μl DAPI for DNA staining in the second wash step. Formamide was washed 3 times in 2x SSC, and samples were resuspended in Imaging Buffer (glucose oxidase buffer with glucose oxidase and catalase, Sigma). For imaging, two coverslips were used to compact samples for improved imaging quality. Imaging was done using a Nikon Ti microscope with a mercury light source (Prior), and images were acquired on a Pixis 1024 (Princeton Instruments) CCD camera. Custom filters for Cy5 (628 nm-40 exciter, 685 nm-40 emitter, 655 nm dichroic) and Alexa594 (590 nm-20, 628 nm-25, 602 nm) were used (Semrock). Exposure times were 2 sec for both filter sets. We collected Z-stacks of 0.3 μm across the sample, 15-25 slices per sample.

smFISH images were processed using custom Matlab (Mathworks) scripts. In essence, images were filtered twice with Laplacian-of-Gaussian edge-detection filters to amplify smFISH signal, and a threshold was defined from the signal intensity histogram as the first plateau that separates background from signal. Only images that could be processed at high quality and clear thresholding were used for tissue-specificity analysis. We used manual inspection of high-quality smFISH images of each KMT probe set to determine the tissues of expression. We scored a gene as being expressed in a tissue if we detected expression in most cells in that

tissue in at least 10 animals. Tissues were identified by position in the animal, and individual cells by nuclear positions and nuclear morphology.

msp mRNA fluorescence was quantified from raw images. Background signal was subtracted and was <1% of the *msp* signal for all genotypes. *msp* expression was quantified in late-stage mature spermatocytes by quantifying the average signal across several cells.

Broodsize assays

For broodsize determination, young L4 animals were individually placed on plates with food and transferred to fresh plates every 24 hr for 3 days. The number of progeny was determined by counting the number of adult animals produced over 6 days. All experiments were performed at 25˚C. Strains were always maintained at 20˚C and only raised to the experimental temperature as young larvae for broodsize analysis.

Single-copy transgene strain construction

Single-copy transgenes were created essentially as described (Frojkaer-Jensen, 2008; 2012). We cloned the genomic sequence of *set-17* from the upstream open reading frame to the stop codon of *set-17, C. elegans* optimized eGFP and the *set-17* 3'UTR and downstream non-coding sequence into the targeting vector for the chromosome *IV* integration site *cxTi10882*. This targeting vector carries the *unc-119(+)* visible marker and two recombination arms of 1.5 kb complementarity to the region of integration into the genome. We chose the Chr. IV site because Frokjaer-Jensen et al. had shown *cxTi10882* to be competent for germline expression. We injected our *set-17::GFP* construct together with the transposase and a negative selection marker expressing the proteotoxin *peel-1* under a heat-shock promoter into *set-17(n5017) II; unc-119(ed3) III; cxTi10882 IV* hermaphrodites (see wormbuilder.com and

Frokjaer-Jensen et al., 2012, for details about the plasmids and procedure). For the construction of a germline-specific *set-17* rescue strain, we replaced the *set-17* 5' upstream region with the *mex-5* minimal promoter and followed the same injection and selection protocol.

Mating assays

Mating efficiency was determined at 25˚C. Individual L4 females (hermaphrodites feminized by *fog-2* mutation) were moved to standard plates for 24 hrs to ensure they had not previously mated. L4 males were also separated from females for 24 hrs prior to mating assays. For mating assays, a single male was placed onto a 6 cm plate with one female. Bacterial lawns on mating plates were kept small, ~1 cm diameter. Mating was allowed to proceed for 24 hrs, after which the male was removed and the female was moved to a new plate every 24 hrs for 4 days, until after all progeny had been generated. We determined separately that *set-17* animals generate 1:1 males:females from crosses (data not shown).

Spermatid counts

To determine the number of spermatids in hermaphrodite spermathecas, we maintained animals at 25˚C and collected bleach-synchronized early young adults. We selected only animals that had completed sperm production and with oocytes that either had loaded into the spermatheca or were about to be loaded. We stained animals with DAPI and imaged them using an inverted Nikon Ti Epi-fluorescence microscope with a 100x 1.4 NA oil objective. We collected Z-stacks of 0.5 µm thickness through the entire spermathecal region (10-20 slices). Images were collected using a Pixis 1024 CCD (Princeton Instruments) or an Orca Flash 4.0 scMOS (Hamamatsu) camera. To quantify spermatids, we counted DNA stained bodies

manually in the Z-stacks. Often only one spermatheca could be imaged per animal because the intestine obstructed reliable quantification of spermatids.

Electron microscopy

Males were maintained at 25˚C and collected 24 hrs post-L4 stage as adults. Adult males were fixed in 0.7% glutaraldehyde, 0.7% OsO_4, 0.1M cacodylate buffer for 1 hr on ice. During this step the head region of individual worms was excised. Worms were washed in 0.1M cacodylate buffer and post-fixed in 2% OsO_4 in 0.1 M cacodylate buffer overnight at 4˚C. Three to five worms were mounted into agar blocks, dehydrated in a series of alcohols and embedded in Epon resin. Thin sections of 50-70 nm of longitudinal and cross sections were obtained using an Ultracut microtome E. Samples were observed using a JEOL JEM 1200 EX II electron microscope at 80 kv and imaged with a side-mounted AMT XR-41 CCD camera.

Images of wild-type and *set-17* adult male germlines were inspected manually for abnormalities of germ cells, spermatocytes or spermatids. The *set-17* defect in fibrous-body membranous organelle production was first detected at this level. Quantification of cytoplasmic cross-sectional area was done manually using Image J and Excel. Cytoplasmic cross-sectional area was defined as the area of the total cell minus that of the nucleus. The size of individual FB-MOs was determined manually by tracing their circumferences. We computed the percent cross-sectional cytoplasmic area of FB-MOs by dividing the sum of the individual FB-MO cross-sectional areas by that of the cytoplasmic cross-sectional area. FB-MOs were identified by homogeneous grey staining and the double-membrane around the grey structures. We analyzed spermatocytes from at least three different adult males of wild-type, *set-17* and *set-17; $P_{set\text{-}17}$::set-17(+)*.

To calculate the number of FB-MOs per cell from the EM cross sections, the diameter of individual FB-MOs has to be taken into account. Since the cross sections randomly sampled the cells, the probability of observing an FB-MO is proportional to its diameter. We computed the radii of FB-MOs assuming that they behave like spheres, i.e. that the radius is proportional to the square root of the area. Using FB-MO areas already determined, we computed the average FB-MO radius for wild-type, *set-17* and *set-17; $P_{set\text{-}17}$::set-17(+)*. We then normalized the observed number of FB-MOs by the observed radii from wild-type, *set-17* and *set-17; $P_{set\text{-}17}$::set-17(+)* adult males.

Special membrane structures in spermatids were quantified by manual inspection of EM cross-sections of spermatids.

Immunofluorescence of male spermatocyte nuclei

We maintained males at 25˚C and extruded their germlines by microsurgery with syringe needles. Fixation and staining were conducted as described (Libuda et al., 2014). Germlines were fixed for 5 min in 1% MeOH-free formaldehyde, frozen and treated with -20˚C MeOH for 1 min. Samples were blocked in 1% BSA, 1x PBST for 45 min and incubated with primary antibodies overnight at 4˚C. Secondary antibody staining followed for 2 hrs at room temperature. Samples were imaged using a Zeiss LSM800 confocal microscope with a 63x oil objective. The following antibodies were used: anti-GFP (mouse monoclonal, Roche), anti-H3K4me1 (rabbit polyclonal, Abcam), anti-H3K4me2 (rabbit polyclonal, Millipore). Secondary: goat anti-mouse (Alexa488, Life Technologies), goat-anti-rabbit (Alexa 594, Life Technologies).

SET-17::GFP foci were quantified manually from Z-stacks collected with Airy-scan resolution (0.1 µm Z-sections).

Transcript analysis using RNA sequencing

We collected total RNA from two biological replicates of bleach-synchronized L4s from wild-type, *set-17(n5017)*, *set-17(n5017) II ; unc-119(ed3) III ; nSi3[P_{set-17}::set-17(+)::GFP::set-17 3'UTR; unc-119(+)] IV* and *set-17(n5017) II ; unc-119(ed3) III ; nSi3[P_{mex-5}::set-17(+)::GFP::set-17 3'UTR; unc-119(+)] IV* L4 hermaphrodites. We prepared RNA sequencing libraries from total RNA using the NEB NextSeq Kit and the protocol described (Wurtzel et al., 2015). Libraries were sequenced using a single lane of an Illumina Hi-Seq by the MIT Biomicro Center. Reads were mapped to the *C. elegans* transcriptome using the RSem package. Differential gene expression analysis between wild-type and *set-17* mutant transcriptomes was conducted using the package EdgeR (Anders et al., 2013; Robinson et al., 2010). A stringent false-discovery-rate (FDR) of < 0.05 was used to identify the 123 transcripts that are high-confidence misregulated transcripts in *set-17* mutants.

We identified spermatocyte-specific genes using data from previous analyses of germline gene expression (Ortiz et al., 2014).

The fold-change enrichment distributions for *set-17*-misregulated genes, *msp* genes or the spermatogenic gene clusters were evaluated based on their cumulative distribution functions (CDF) and non-parametric CDF-based statistical tests, Mann-Whitney (MW) and Kolmogorov-Smirnov (KS). Such tests were appropriate given the asymmetric negative skew of the fold-change distributions.

Identification of clusters of spermatocyte-enriched genes

Using the chromosome and base-pair start position of all previously identified spermatogenic and oogenic genes in WormMine (wormbase.org, genome assembly WS210), we generated histograms with a bin size of 50 kb to identify gene clusters of spermatogenic and/or oogenic genes. To test for significant enrichment of genes in a particular 50 kb interval we used the hypergeometric test as described (Miller et al., 2004), with a multiple-hypothesis-testing correction for the number of spermatogenic genes per chromosome. We chose a cut-off of FDR < 0.05 for cluster identification for all chromosomes.

Transcription site (TS) analysis

msp transcription sites (TSs) were identified in the nuclei of primary spermatocytes that also exhibited *msp* mRNA expression in the cytoplasm. Foci were visually separated from background using a finite Fourier transform bandpass filter to suppress high frequencies and counted manually. Nuclei were identified based on DNA staining with DAPI.

To determine the probability of transcriptional activity of individual *msp* gene clusters, we tested if the TS data followed the binomial distribution. The TS distribution should be governed by a simple binomial model if TSs are independent random events that occur with equal probability at each cluster. The probability of cluster activation is then given by the empirical mean number of clusters per nucleus divided by the number of possible states. The number of states, i.e. the maximal number of *msp* gene clusters that were transcriptionally active simultaneously was equal to four in our experiments.

To visualize SET-17::GFP and *msp* TSs, we prepared samples as described above for smFISH imaging, except we used EM-grade MeOH-free formaldehyde and fixed samples in the

dark to detect GFP fluorescence from the transgene directly without additional labeling of the protein. To preserve GFP fluorescence we identified *msp* transcription sites in the nuclei of *msp*-expressing spermatocytes and imaged SET-17::GFP only at those sites. We computed the fraction of *msp* transcription sites that also were SET-17::GFP foci.

Fluorescence recovery after photo-bleaching (FRAP)

We immobilized 1-day old adult males with 10 mM levamisole (an acetylcholine agonist that immobilizes animals by inducing muscle contraction) on soft 2% agarose pads for confocal imaging. Animals can be revived from this preparation for up to 1 hr after immobilization. We imaged spermatocytes that were proximal to the cover slip, i.e. only the hypoderm and cover slip separated the objective and the spermatocytes. We identified imaging regions of interest of different sizes, including at least two spermatocyte nuclei for each FRAP experiment (one for FRAP measurements, one for bleaching and movement control). We imaged several Z-slices of 0.5-1 µm separation, because there was frequent shifting of the animals, altering the focal plane of the foci. A region of interest for FRAP was defined manually for a given SET-17::GFP focus. To achieve complete bleaching of GFP in the foci, bleaching was conducted with 25 rounds of 100% laser induction with a 488 nm laser (Zeiss LSM 700). Spermatocytes were then tracked for at least 6 min, up to 20 min.

Data were analyzed manually, tracking the bleached ROI in the movies and quantifying average fluorescence for the bleached and control foci.

FRAP traces were normalized to the focus fluorescence detected in the ROI after bleaching (floor) and to the fluorescence before bleaching (maximal). Recovery traces were

individually fitted with single exponential association curves and the half-maximal recovery times for each trace were averaged.

Supplemental Figure Legends

Figure S1

(A) Similarity tree based on the pairwise alignment scores of all *H. sapiens* and *C. elegans* SET-domains.

(B) Representative images of smFISH labeling of *set-17* and *set-2* mRNAs, respectively, in the germline of a wild-type adult hermaphrodite. Maximum projection of 45 Z-slices. Scale bar, 20 µm. The grey area covers an image processing artifact.

Figure S2

(A) Above: Gene model of *set-17* with the SET-domain and mutations used indicated. Coding sequence, black; SET-domain, red; thin lines, introns. Scale bar, 100 bp. Below: Protein domain structures of *H. sapiens* PRDM9 and *C. elegans* SET-17. Amino acid percent identity determined by ClustalW alignment. N, N-terminus; C, C-terminus.

(B) Full-length protein domain structure of SET-domain proteins from *C. elegans* and *H. sapiens*, ordered by similarity of their SET-domains and germline expression in the respective organism is indicated. Red: PRDM9/7-family SET-domain; Green: PRDM1-family SET-domain; Orange: Clr4-family SET-domain. The alignment distances are qualitative. Germline expression of PRDM7 was detected by sequencing of whole human tissues and PRDM7 expression is enriched in testes (GTEx portal, Illumina Body Map). SET-17 is more similar to PRDM7 and PRDM9 than to any of the other SET-domain proteins. *C. elegans* BLMP-1 is the ortholog of *H. sapiens* PRDM1 and neither is expressed in the germline. SET-17 and BLMP-1 are the only PRDM-family SET-domains in *C. elegans* (see Fig S1A). The SET-domain of *C. elegans* SET-11 is least similar to the PRDM-type SET-domains. SET-domain identity between PRDM9 and SET-17: 48%; SET-domain identity between PRDM7 and SET-17: 47%; SET-domain identity between PRDM9 and PRDM7 97%; BLMP-1 and PRDM1: 41%; SET-17 and PRDM1: 38%; SET-17 and BLMP-1: 30%; SET-11 and PRDM9: 25%; SET-11 and PRDM1: 20%; SET-11 and SET-17: 23%; SET-11 and BLMP-1: 15%. (% identity obtained with ClustalW, SET-domain sequences from Uniprot)

(C) Broodsizes of *set-17* and *set-17* double mutants with select germline-expressed KMTs. n > 15.

Figure S3

(A) Number of FB-MOs per primary spermatocyte in wild type, *set-17* and *set-17; $P_{set\text{-}17}$::set-17(+)* adult male, corrected for mean FB-MO size and cross-sectional area. n = 10; * $P < 0.05$, t-test.

(B) Quantification of special membrane structures (SMS) in mature spermatids of wild-type, *set-17* and *set-17; $P_{set\text{-}17}$::set-17(+)* adult males. Arrowheads, representative SMS. n > 18. Scale bar, 500 nm.

Figure S4

(A) Numbers of SET-17::GFP foci per spermatocyte nucleus in early, mid and late primary spermatocytes in adult males. Karyo = karyosome, nuclei that are undergoing transformation to secondary spermatocytes.

(B) Confocal image of SET-17::GFP in spermatocytes of a live immobilized L4 hermaphrodite. Representative Z-section. Scale bar, 5 µm.

(C) Confocal image of SET-17::GFP in the oocyte-producing germline of a live immobilized adult hermaphrodite. Representative Z-section. Scale bar, 5 µm.

(D) Confocal image of SET-17::GFP in the hypoderm of a live immobilized adult hermaphrodite. Representative Z-section. Scale bar, 5 µm.

(E), (F) Immunostaining of primary spermatocyte nuclei from an adult male expressing SET-17::GFP as in Fig. 4C & 4D. Nuclei are stained for (E) H3K4me1 and (F) H3K4me2, as well as SET-17::GFP. DNA stained by DAPI.

Figure S5

(A) Summary of transcriptome analysis of expression in wild-type and *set-17* mutants. logFC: logarithm (base 2) of the fold-change *set-17* / wild-type; logCPM: logarithm (base 2) of the counts per million, a measure of expression level of a transcript. The 123 transcripts that were identified as significantly different between *set-17* and wild-type are indicated in red (see methods, EdgeR).

(B) Cumulative distribution of the fold-change over wild-type values for the 123 significantly misregulated genes in *set-17*, plotted for *set-17* and the two rescue lines expressing wild-type *set-17* from its endogenous promoter (*set-17; $P_{set\text{-}17}$::set-17(+)*, orange) or a germline-specific promoter (*set-17; $P_{mex\text{-}5}$::set-17(+),* yellow), compared with all 2306 previously identified spermatogenic transcripts in *set-17* (black).

(C) Rank-correlation analysis of the spermatogenic gene enrichment in the 123 significantly misregulated transcripts in *set-17* mutants. Plotted here is the cumulative fraction of genes that are spermatogenic for a given rank (the distribution drops for every non-spermatogenic gene in the list of 123 misregulated genes).

(D) The relative average levels of expression of all 28 *msp* genes for the indicated genotypes, based on the RPKM values of individual *msp* genes. These are the same data as in Fig. 5C but depicted as a percentage rather than a log ratio.

(E) Levels of *msp* expression of all 28 *msp* genes correlate with *set-17* transcript levels as measured by RNAseq in wild-type, *set-17*, *set-17; $P_{set\text{-}17}$::set-17(+)* (orange) and *set-17; $P_{mex\text{-}5}$::set-17(+)* (yellow) L4 hermaphrodites.

(F) Broodsizes correlate with *set-17* transcript levels as measured by RNAseq in wild-type, *set-17*, *set-17; $P_{set\text{-}17}$::set-17(+)* (orange) and *set-17; $P_{mex\text{-}5}$::set-17(+)* (yellow) L4 hermaphrodites.

(G) Cumulative distribution of the fold-change vs. wild-type values from RNAseq studies of L4 hermaphrodites for the 72 genes in the spermatogenic gene cluster on chromosome *II* in *set-17* (blue), *set-17; $P_{set\text{-}17}$::set-17(+)* (orange) and *set-17; $P_{mex\text{-}5}$::set-17(+)* (yellow) and for the 365 spermatogenic genes on chromosome *II* not in the cluster in *set-17* (grey). These data are the same as in Fig. 5F. These distributions were used to calculate statistical significance using non-parametric tests.

(H) Cumulative distribution of the fold-change vs. wild-type values from RNAseq studies of L4 hermaphrodites for the 176 genes in the two spermatogenic gene clusters on chromosome *IV* (Cl. IVA+B) in *set-17* (blue), *set-17; $P_{set\text{-}17}$::set-17(+)* (orange) and *set-17; $P_{mex\text{-}5}$::set-17(+)* (yellow) and for the 328 spermatogenic genes on chromosome *IV* not in the cluster in *set-17* (grey). These data are the same as in Fig. 5G. These distributions were used to calculate statistical significance using non-parametric tests.

(I) Cumulative distribution of the fold-change vs. wild-type values from RNAseq studies of L4 hermaphrodites for the 72 genes in the spermatogenic gene cluster on chromosome *II* in *set-17* - minus the 12 *msp* genes (blue) and for the 328 spermatogenic genes on chromosome *IV* not in the cluster in *set-17* (grey). $P < 0.0001$, MW or KS test.

(J) Cumulative distribution of the fold-change over wild-type values from RNAseq studies of L4 hermaphrodites for the 176 genes in the two spermatogenic gene clusters on chromosome *IV* (Cl. IVA+B) in *set-17* minus the 16 *msp* genes (blue) and for the 328

spermatogenic genes on chromosome *IV* not in the cluster in *set-17* (grey). $P < 0.0001$, MW or KS test.

(K) Histogram of spermatogenic genes on chromosome *V* in 50 kb bins. We identified a previously uncharacterized sperm-gene cluster around position 1.5×10^7 bp. $P < 0.0001$, hypergeometric test with FDR correction.

Figure S6

(A) Empirical transcription site distribution of *msp* genes in primary spermatocytes for wild-type and *set-17* as in Fig. 6F. Dotted lines indicate the simulated distribution of TSs generated by a binomial model using the following parameters derived from the empirical data. Number of states, the observed maximum number of states in either the wild type or *set-17* was equal to four; mean, empirical means of the observed distributions of TSs in wild type and *set-17* (respectively, solid lines) normalized by four, the maximum number of states observed.

(B) Goodness-of-fit analysis of the simulated and the empirical data in Fig. S6A showing the simulated data as a function of the empirical data and examining their correlation. Wild-type, R^2 = 0.853; *set-17*, R^2 = 0.987.

(C) Above: frames of a confocal movie showing the fusion of two SET-17::GFP foci in the nucleus of a primary spermatocyte in an immobilized adult male. Scale bar, 1 µm. Below: Quantification of SET-17::GFP foci signal intensity over time, showing the sudden increase in fluorescence of one focus, while another focus stayed constant.

(D) Above: frames of a confocal movie showing the fission of a SET-17::GFP focus in the nucleus of a primary spermatocyte in an immobilized adult male. Scale bar, 1 µm. Below: Quantification of SET-17::GFP focus signal intensity over time, showing the sudden decrease in fluorescence of the focus.

Figure S7

(A) Histogram of the positions of the predicted *elt-1* target genes on chromosome *II* in 50 kb bins (purple), plotted with the distribution of positions of all spermatogenic genes (grey) and all *msp* genes (yellow) on chromosome *II*. The spermatogenic gene cluster is indicated in light blue.

(B) Histogram of the positions of the predicted *elt-1* target genes on chromosome *IV* in 50 kb bins (purple), plotted with the distribution of positions of all spermatogenic genes (grey) and all *msp* genes (yellow) on chromosome *IV*. The spermatogenic gene clusters are indicated in light blue.

(C) Fertility (No. progeny % wild type) plotted as a function of *msp* expression (average *msp* RPKM or as *msp* FISH % wild type), data combined from Fig. S5F and Fig. 7F. $R^2 = 0.974$.

(D) Representative confocal images of SET-17::GFP in spermatocytes of a live immobilized L4 hermaphrodite treated with RNAi against *elt-1, unc-22* (Ctrl) or *gfp*, respectively. RNAi against *elt-1* did not affect SET-17::GFP foci.

(E) Overlaps between (1) all 445 genes misexpressed in progressively sterile *spr-5* adult hermaphrodites and the 123 genes misregulated in *set-17* L4 hermaphrodites, (2) all 445 genes misexpressed in progressively sterile *spr-5* adult hermaphrodites and the 60 *set-17* spermatogenic genes misexpressed in L4 hermaphrodites and (3) the 202 spermatogenic genes misexpressed in progressively sterile *spr-5* adult hermaphrodites and the 60 *set-17* spermatogenic genes misexpressed in L4 hermaphrodites. *spr-5* gene expression data from Katz et al. (2009); spermatogenic gene expression categories from Ortiz et al. (2014). P < 1E-20 for each category, hypergeometric test.

Supplementary Table 1

Summary of endogenous KMT mRNAs detected in the germline of L1 and wild-type adult hermaphrodites. Blue, expressed in primordial germ cells; purple, expressed throughout the adult hermaphrodite germline; yellow, expressed specifically in oocytes in the adult hermaphrodite germline, but not in the primordial germ cells.
