# Supplementary figures and images for: A Caenorhabditis elegans protein with a PRDM9-like SET domain localizes to chromatin-associated foci and promotes spermatocyte gene expression, sperm production and fertility
Source: PLoS Genet. 2018 Apr 27;14(4):e1007295. doi: 10.1371/journal.pgen.1007295 (PMC5942854; doi:10.1371/journal.pgen.1007295)

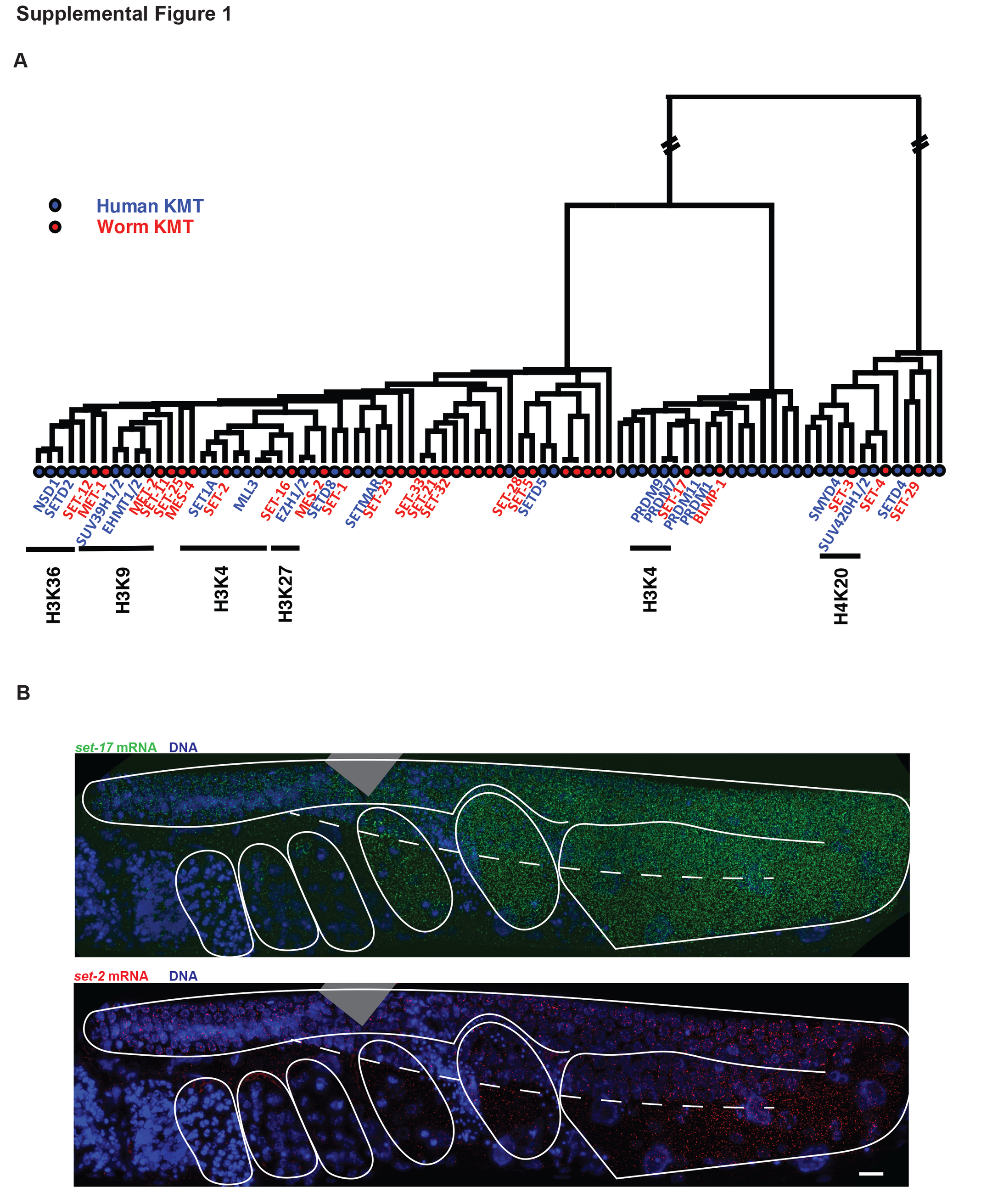

Supplement: S1 Fig — A) Similarity tree based on the pairwise alignment scores of all H. sapiens and C. elegans SET-domains. B) Representative images of smFISH labeling of set-17 and set-2 mRNAs, respectively, in the germline of a wild-type adult hermaphrodite. Maximum projection of 45 Z-slices. Scale bar, 20 μm. The grey area covers an image processing artifact. (TIF) [file pgen.1007295.s003.tif]

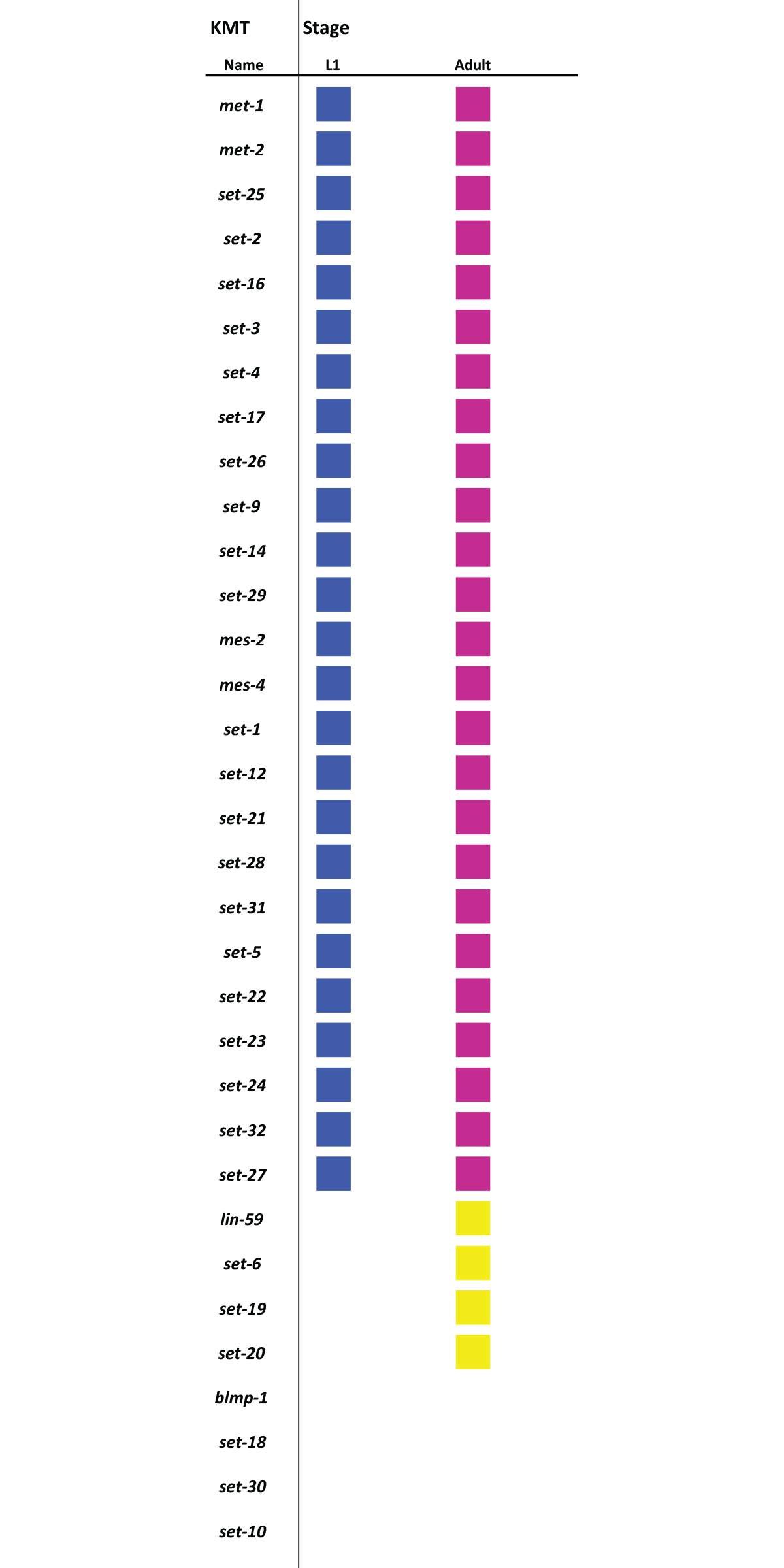

Supplement: S2 Fig — Blue, expressed in primordial germ cells; purple, expressed throughout the adult hermaphrodite germline; yellow, expressed specifically in oocytes in the adult hermaphrodite germline, but not in the primordial germ cells. (TIF) [file pgen.1007295.s004.tif]

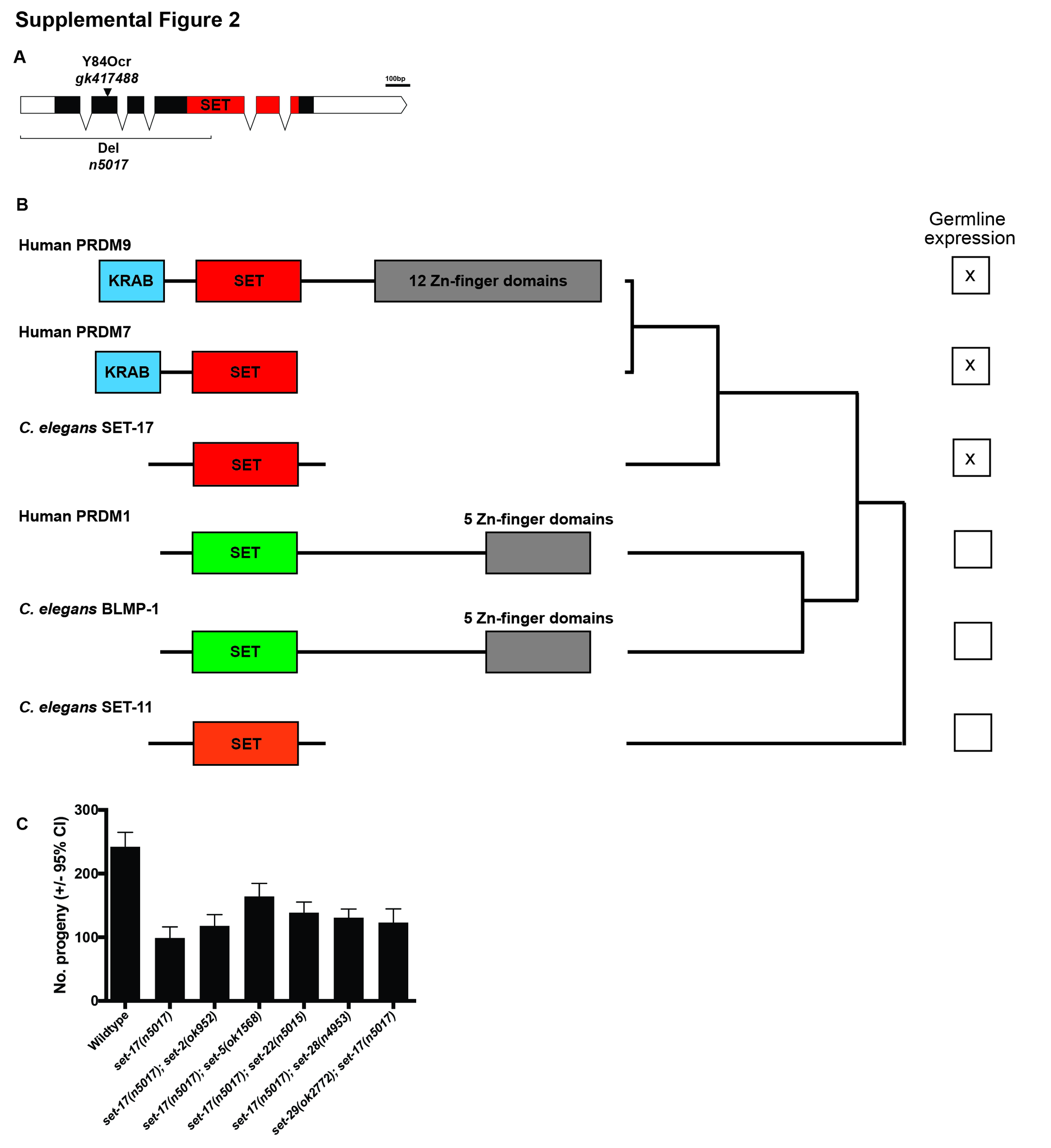

Supplement: S3 Fig — A) Above: Gene model of set-17 with the SET-domain and mutations used indicated. Coding sequence, black; SET-domain, red; thin lines, introns. Scale bar, 100 bp. Below: Protein domain structures of H. sapiens PRDM9 and C. elegans SET-17. Amino acid percent identity determined by ClustalW alignment. N, N-terminus; C, C-terminus. B) Full-length protein domain structure of SET-domain proteins from C. elegans and H. sapiens, ordered by similarity of their SET-domains and germline expression in the respective organism is indicated. Red: PRDM9/7-family SET-domain; Green: PRDM1-family SET-domain; Orange: Clr4-family SET-domain. The alignment distances are qualitative. Germline expression of PRDM7 was detected by sequencing of whole human tissues and PRDM7 expression is enriched in testes (GTEx portal, Illumina Body Map). SET-17 is more similar to PRDM7 and PRDM9 than to any of the other SET-domain proteins. C. elegans BLMP-1 is the ortholog of H. sapiens PRDM1 and neither is expressed in the germline. SET-17 and BLMP-1 are the only PRDM-family SET-domains in C. elegans (see S1A Fig). The SET-domain of C. elegans SET-11 is least similar to the PRDM-type SET-domains. SET-domain identity between PRDM9 and SET-17: 48%; SET-domain identity between PRDM7 and SET-17: 47%; SET-domain identity between PRDM9 and PRDM7 97%; BLMP-1 and PRDM1: 41%; SET-17 and PRDM1: 38%; SET-17 and BLMP-1: 30%; SET-11 and PRDM9: 25%; SET-11 and PRDM1: 20%; SET-11 and SET-17: 23%; SET-11 and BLMP-1: 15%. (% identity obtained with ClustalW, SET-domain sequences from Uniprot). C) Broodsizes of set-17 and set-17 double mutants with select germline-expressed KMTs. n > 15. (TIF) [file pgen.1007295.s005.tif]

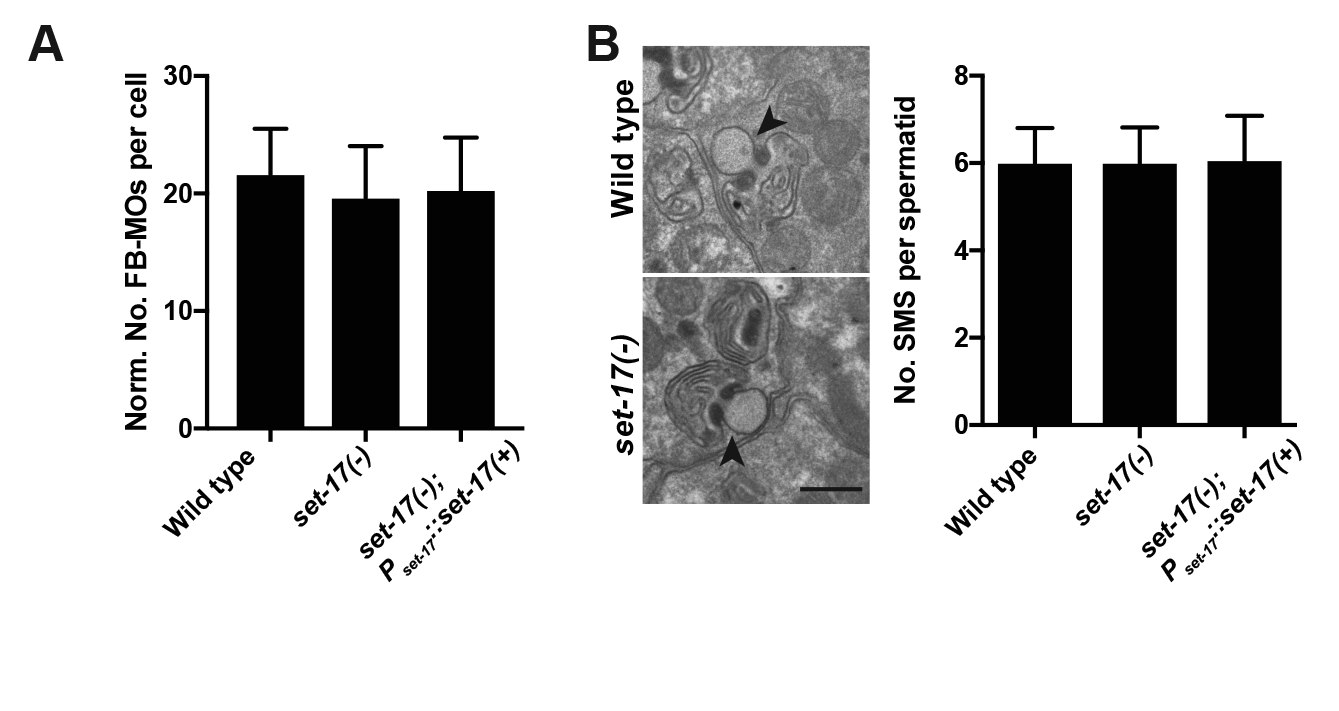

Supplement: S4 Fig — A) Number of FB-MOs per primary spermatocyte in wild type, set-17 and set-17; Pset-17::set-17(+) adult male, corrected for mean FB-MO size and cross-sectional area. n = 10; * P < 0.05, t-test. B) Quantification of special membrane structures (SMS) in mature spermatids of wild-type, set-17 and set-17; Pset-17::set-17(+) adult males. Arrowheads, representative SMS. n > 18. Scale bar, 500 nm. (TIF) [file pgen.1007295.s006.tif]

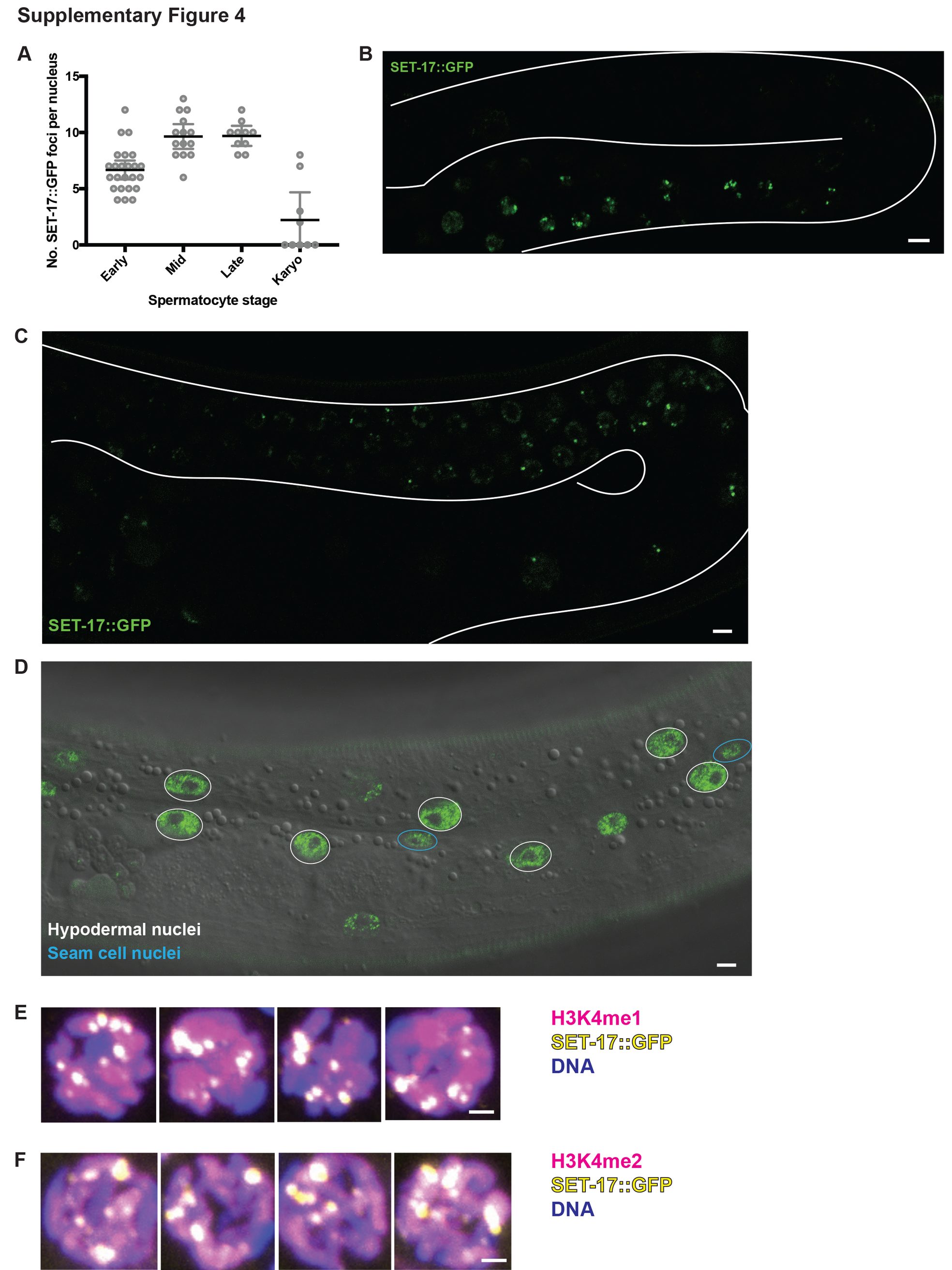

Supplement: S5 Fig — A) Numbers of SET-17::GFP foci per spermatocyte nucleus in early, mid and late primary spermatocytes in adult males. Karyo = karyosome, nuclei that are undergoing transformation to secondary spermatocytes. B) Confocal image of SET-17::GFP in spermatocytes of a live immobilized L4 hermaphrodite. Representative Z-section. Scale bar, 5 μm. C) Confocal image of SET-17::GFP in the oocyte-producing germline of a live immobilized adult hermaphrodite. Representative Z-section. Scale bar, 5 μm. D) Confocal image of SET-17::GFP in the hypoderm of a live immobilized adult hermaphrodite. Representative Z-section. Scale bar, 5 μm. E) & F) Immunostaining of primary spermatocyte nuclei from an adult male expressing SET-17::GFP as in Fig 4C & 4D. Nuclei are stained for H3K4me1 (E) and H3K4me2 (F), as well as SET-17::GFP. DNA stained by DAPI. (TIF) [file pgen.1007295.s007.tif]

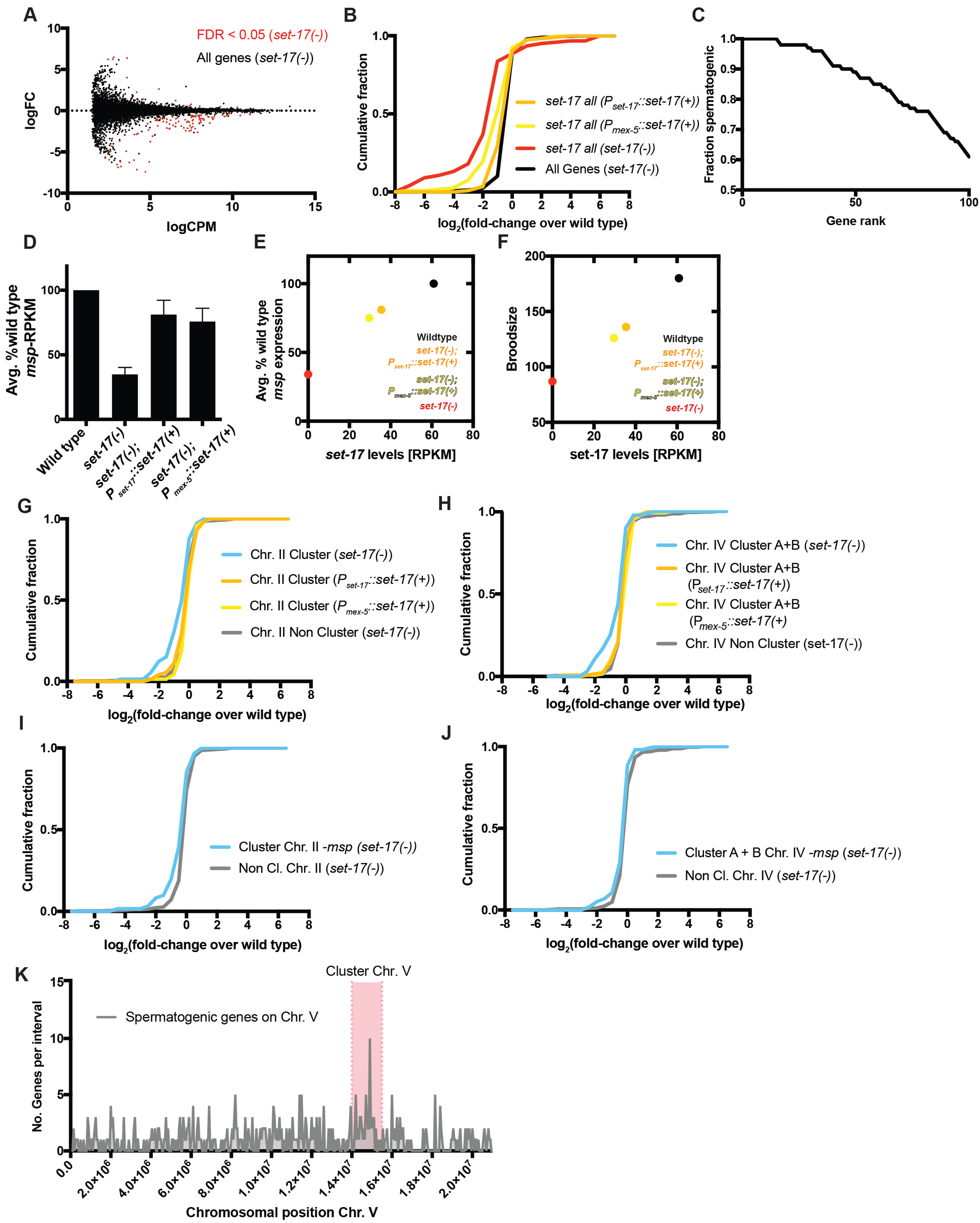

Supplement: S6 Fig — A) Summary of transcriptome analysis of expression in wild-type and set-17 mutants. logFC: logarithm (base 2) of the fold-change set-17 / wild-type; logCPM: logarithm (base 2) of the counts per million, a measure of expression level of a transcript. The 123 transcripts that were identified as significantly different between set-17 and wild-type are indicated in red (see methods, EdgeR). B) Cumulative distribution of the fold-change over wild-type values for the 123 significantly misregulated genes in set-17, plotted for set-17 and the two rescue lines expressing wild-type set-17 from its endogenous promoter (set-17; Pset-17::set-17(+), orange) or a germline-specific promoter (set-17; Pmex-5::set-17(+), yellow), compared with all 2306 previously identified spermatogenic transcripts in set-17 (black). C) Rank-correlation analysis of the spermatogenic gene enrichment in the 123 significantly misregulated transcripts in set-17 mutants. Plotted here is the cumulative fraction of genes that are spermatogenic for a given rank (the distribution drops for every non-spermatogenic gene in the list of 123 misregulated genes). D) The relative average levels of expression of all 28 msp genes for the indicated genotypes, based on the RPKM values of individual msp genes. These are the same data as in Fig 5C but depicted as a percentage rather than a log ratio. E) Levels of msp expression of all 28 msp genes correlate with set-17 transcript levels as measured by RNAseq in wild-type, set-17, set-17; Pset-17::set-17(+) (orange) and set-17; Pmex-5::set-17(+) (yellow) L4 hermaphrodites. F) Broodsizes correlate with set-17 transcript levels as measured by RNAseq in wild-type, set-17, set-17; Pset-17::set-17(+) (orange) and set-17; Pmex-5::set-17(+) (yellow) L4 hermaphrodites. G) Cumulative distribution of the fold-change vs. wild-type values from RNAseq studies of L4 hermaphrodites for the 72 genes in the spermatogenic gene cluster on chromosome II in set-17 (blue), set-17; Pset-17::set- [file pgen.1007295.s008.tif]

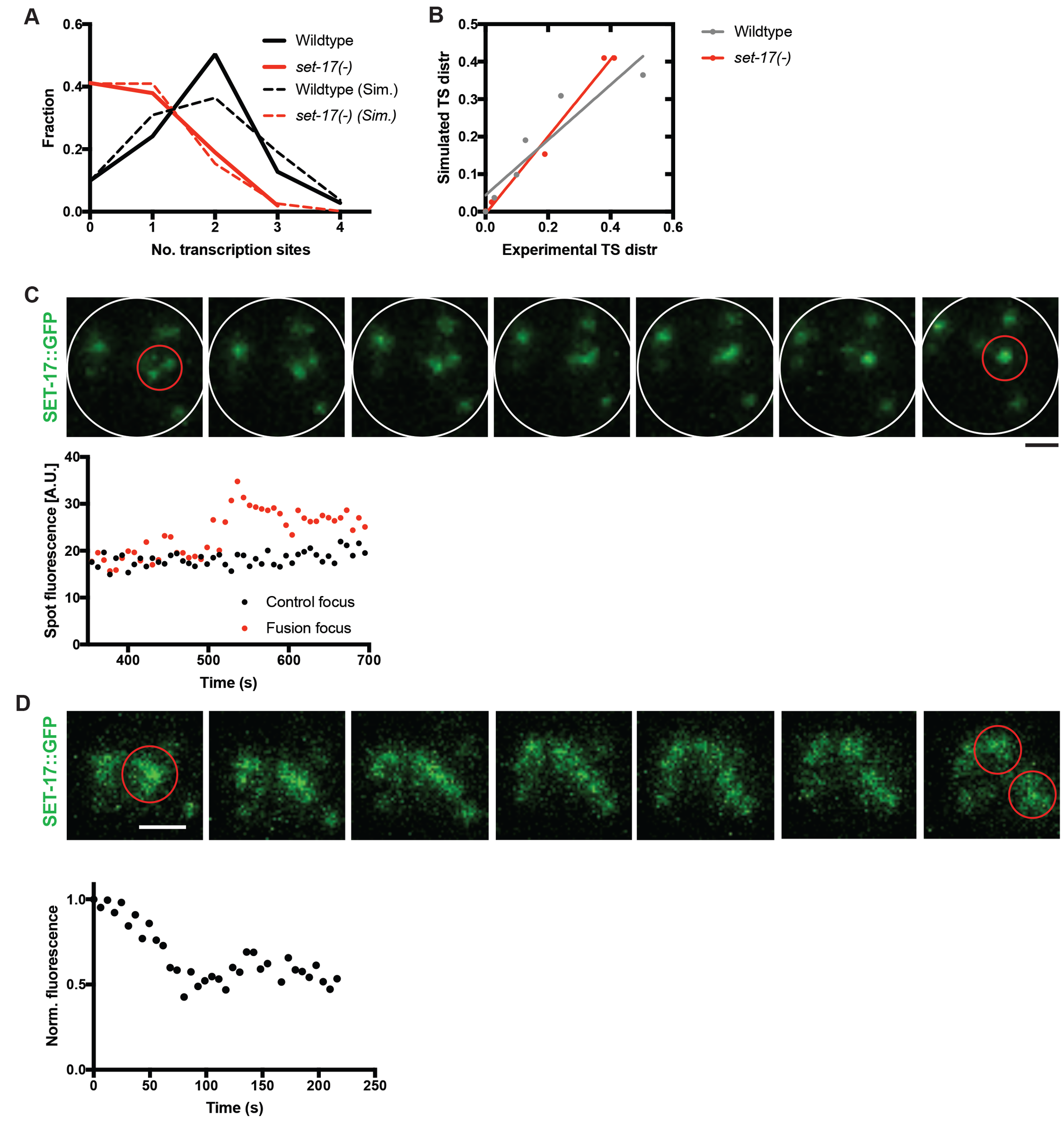

Supplement: S7 Fig — A) Empirical transcription site distribution of msp genes in primary spermatocytes for wild-type and set-17 as in Fig 6F. Dotted lines indicate the simulated distribution of TSs generated by a binomial model using the following parameters derived from the empirical data. Number of states, the observed maximum number of states in either the wild type or set-17 was equal to four; mean, empirical means of the observed distributions of TSs in wild type and set-17 (respectively, solid lines) normalized by four, the maximum number of states observed. B) Goodness-of-fit analysis of the simulated and the empirical data in S6A Fig showing the simulated data as a function of the empirical data and examining their correlation. Wild-type, R2 = 0.853; set-17, R2 = 0.987. C) Above: frames of a confocal movie showing the fusion of two SET-17::GFP foci in the nucleus of a primary spermatocyte in an immobilized adult male. Scale bar, 1 μm. Below: Quantification of SET-17::GFP foci signal intensity over time, showing the sudden increase in fluorescence of one focus, while another focus stayed constant. D) Above: frames of a confocal movie showing the fission of a SET-17::GFP focus in the nucleus of a primary spermatocyte in an immobilized adult male. Scale bar, 1 μm. Below: Quantification of SET-17::GFP focus signal intensity over time, showing the sudden decrease in fluorescence of the focus. (TIF) [file pgen.1007295.s009.tif]

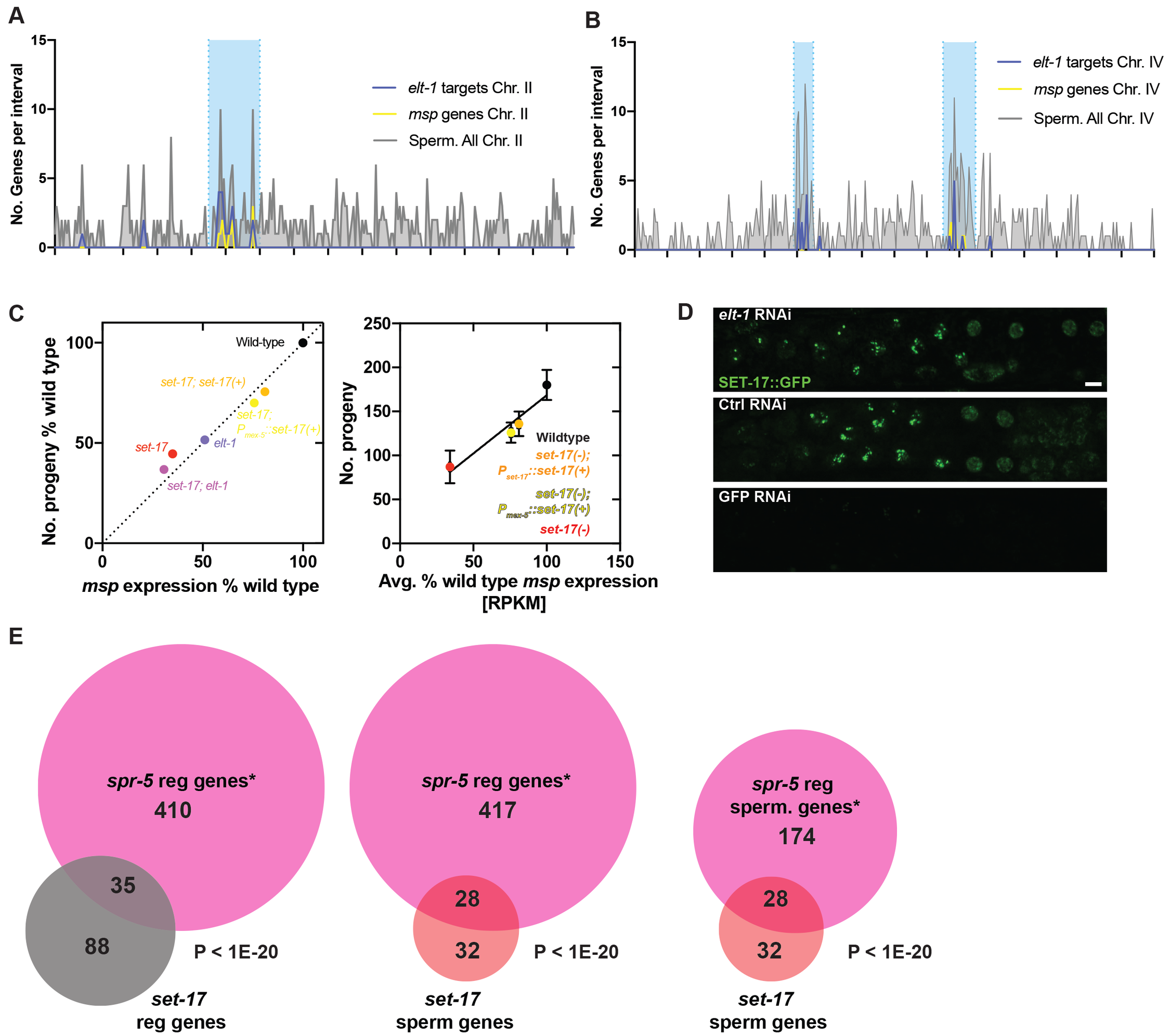

Supplement: S8 Fig — A) Histogram of the positions of the predicted elt-1 target genes on chromosome II in 50 kb bins (purple), plotted with the distribution of positions of all spermatogenic genes (grey) and all msp genes (yellow) on chromosome II. The spermatogenic gene cluster is indicated in light blue. B) Histogram of the positions of the predicted elt-1 target genes on chromosome IV in 50 kb bins (purple), plotted with the distribution of positions of all spermatogenic genes (grey) and all msp genes (yellow) on chromosome IV. The spermatogenic gene clusters are indicated in light blue. C) Fertility (No. progeny % wild type) plotted as a function of msp expression (average msp RPKM or as msp FISH % wild type), data combined from S5F Fig and Fig 7F. R2 = 0.974. D) Representative confocal images of SET-17::GFP in spermatocytes of a live immobilized L4 hermaphrodite treated with RNAi against elt-1, unc-22 (Ctrl) or gfp, respectively. RNAi against elt-1 did not affect SET-17::GFP foci. E) Overlaps between (1) all 445 genes misexpressed in progressively sterile spr-5 adult hermaphrodites and the 123 genes misregulated in set-17 L4 hermaphrodites, (2) all 445 genes misexpressed in progressively sterile spr-5 adult hermaphrodites and the 60 set-17 spermatogenic genes misexpressed in L4 hermaphrodites and (3) the 202 spermatogenic genes misexpressed in progressively sterile spr-5 adult hermaphrodites and the 60 set-17 spermatogenic genes misexpressed in L4 hermaphrodites. spr-5 gene expression data from Katz et al. (2009); spermatogenic gene expression categories from Ortiz et al. (2014). P < 1E-20 for each category, hypergeometric test. (TIF) [file pgen.1007295.s010.tif]
